# Supplementary material for: Competitive protein recruitment in artificial cells
Source: Commun Chem. 2024 Jun 28;7:148. doi: 10.1038/s42004-024-01229-9 (PMC11213860; doi:10.1038/s42004-024-01229-9)
Supplement: Supplementary file 2 — Supplementary Information [file 42004_2024_1229_MOESM2_ESM.pdf]

Supplementary Information

Competitive protein recruitment in artificial cells

Thijs W. van Veldhuisen, Madelief A. M. Verwiel, Sebastian Novosedlik, Luc Brunsveld*, Jan C. M. van Hest*

Supplementary Methods

Synthesis of amylose derivatives

Quaternized amylose (Q-Am), carboxymethylated amylose (Cm-Am), and nitrilotriacetic acid-modified amylose (NTA-Am) were synthesized using procedures based on those previously published.[1,2] The synthetic routes for amylose derivatives are shown in Supplementary Scheme 1. For Q-Am, 12–16 kDa amylose (Carbosynth, 1.5 g) and NaOH (2.8 g) were dissolved in Milli-Q (14.25 mL) at 35 °C. After complete dissolution of the amylose, 3-chloro-2-hydroxypropyltrimethylammonium chloride solution (11.64 mL, 60 wt% in water) was added dropwise into the stirring reaction mixture, which was subsequently stirred overnight at 35 °C. Next, the mixture was neutralized with acetic acid and precipitated into cold ethanol (200 mL). The resulting precipitate was re-dissolved in Milli-Q water and dialyzed extensively against water using regenerated cellulose dialysis tubing (Spectrum Labs, USA) with a 3.5 kDa MWCO before lyophilization. This yielded Q-Am as colorless solids (5 g, ca. 80 % yield), with a degree of substitution of 0.8 as determined by ^{1}H NMR (D_2O) in Supplementary Data 1.

In the case of Cm-Am, 12–16 kDa amylose (Carbosynth, 1.5 g) and NaOH (3.6 g) were dissolved in Milli-Q (15 mL) at 35 °C. After complete dissolution of the amylose, chloroacetic acid (2.7 g) was added dropwise into the stirring reaction mixture, which was subsequently stirred for 2 h at 35 °C. Next, the mixture was neutralized with acetic acid and precipitated into cold ethanol (200 mL). The resulting precipitate was re-dissolved in Milli-Q water and dialyzed extensively against water using regenerated cellulose dialysis tubing (Spectrum Labs, USA) with a 3.5 kDa MWCO before lyophilization. This yielded Cm-Am as colorless solids (5 g, ca. 80% yield), with a degree of substitution of 0.4 as determined by ^{1}H NMR (D_2O) in Supplementary Data 1.

Nitrilotriacetic acid-modified amylose (NTA-Am) was prepared via EDC/NHS activation of the Cm-Am carboxylic acid (Supplementary Scheme 1), followed by amide bond formation with an amine-functionalized NTA. First, Cm-Am (85 mg, 0.39 mmol eq.) was dissolved in 10 mM $NaHPO_4$ buffer (10 mL) adjusted to pH 6 with 1 M HCl. To this was added *N*-hydroxysuccinimide (67 mg, 0.58 mmol) and 1-ethyl-3-(3-dimethylaminopropyl)carbodiimide (222 mg, 1.16 mmol). The reaction mixture was then stirred for 2 h at room temperature. This mixture was subsequently concentrated using 3 kDa MWCO spin filters, diluted with 10 mM $NaHPO_4$ buffer (adjusted to pH 8), and concentrated again to remove reagents and change the pH of the reaction medium for the next step. This centrifuge/dilution cycle was repeated a further two times. For the conjugation of the NTA group, *Nα,Nα*-bis(carboxymethyl)-L-lysine hydrate (152 mg, 0.58 mmol) was first dissolved in 10 mM $NaHPO_4$ buffer (18 mL) with 5% DMSO and adjusted to pH 8. To this solution was added the NHS-activated Cm-Am, and the reaction mixture was left to stir at room temperature overnight. The reaction mixture was concentrated, dialyzed extensively against MilliQ water, and lyophilized to yield NTA-Am (160 mg, ca. 90%) as colorless solids with a degree of substitution of NTA groups of 0.09 as determined by ^{1}H NMR (D_2O) in Supplementary Data 1. In the ^{1}H NMR spectrum, signals corresponding to common EDC/NHS side reactions were observed; signals for β-alanine formation by NHS ring opening and signals for N-acylurea groups were found.[3]

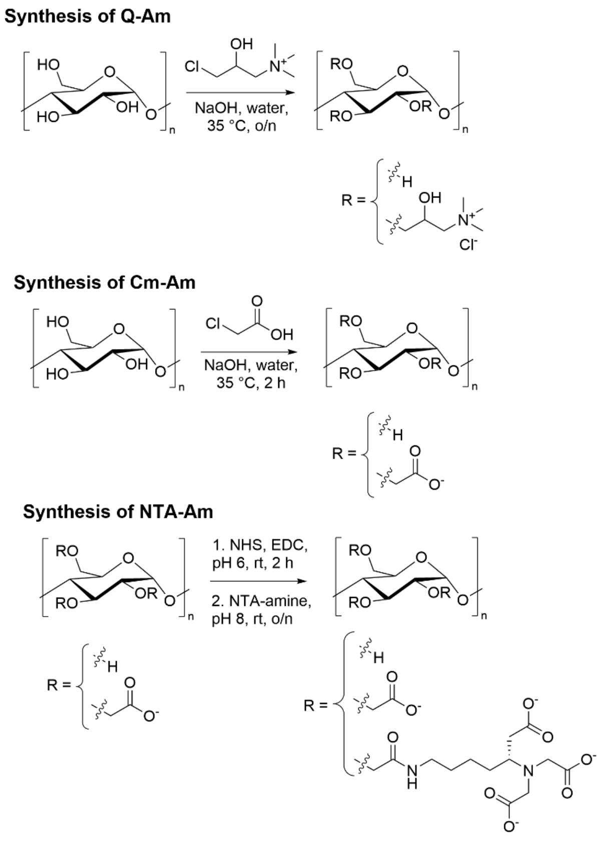

Supplementary Scheme 1. Synthetic routes for amylose derivatives used in this work.

Synthesis of Cy5-labeled Cm-Am

Cy5-labeled Cm-Am was prepared via EDC/NHS activation of the Cm-Am carboxylic acid (Supplementary Scheme 2), followed by amide bond formation with 3-azido-1-propanamine. First, Cm-Am (50 mg, 0.23 mmol eq.) was dissolved in 10 mM MES buffer (10 mL) at pH 6. To this was added *N*-hydroxysulfosuccinimide as a sodium salt (74 mg, 0.34 mmol) and 1-ethyl-3-(3-dimethylaminopropyl)carbodiimide (129 mg, 0.68 mmol). The reaction mixture was then stirred for 2 h at room temperature. This mixture was subsequently concentrated using 3 kDa MWCO spin filters, diluted with 10 mM $NaHPO_4$ buffer (adjusted to pH 8), and concentrated again to remove reagents and change the pH of the reaction medium for the next step. This centrifuge/dilution cycle was repeated a further two times. Next, 3-azido-1-propanamine (45 μL, 0.46 mmol) was added to the Cm-Am solution, and the pH was adjusted to pH 8. The reaction mixture was left to stir at room temperature overnight. The reaction mixture was concentrated, dialyzed extensively against MilliQ water, and lyophilized to yield N_3/Cm-Am (ca. 80% yield) as colorless solids. The amide coupling was confirmed to be successful as determined by ^{1}H NMR (D_2O) in Supplementary Data 1. In the ^{1}H NMR spectrum, signals corresponding to the alkyl groups of the azidopropanamide group were found at 1.8 and 3.4 ppm. Also, a product of an EDC/NHS side reaction was observed in the NMR spectrum: N-acylurea formation.[3] Next, the N_3/Cm-Am (12 mg, 0.86 μmol estimated azide groups) was dissolved in 40 μL of MilliQ, to which was added 10.2 μL of a DMF solution of DBCO-Cy5 (10 mg/mL, 0.086 μmol). The reaction was stirred overnight at room temperature and dialyzed extensively against 20 v/v% DMF in MilliQ water and subsequently 100% MilliQ water, and lyophilized to yield Cy5-labeled Cm-Am as blue fluffy solids (5 mg, 42% yield) without further characterization.

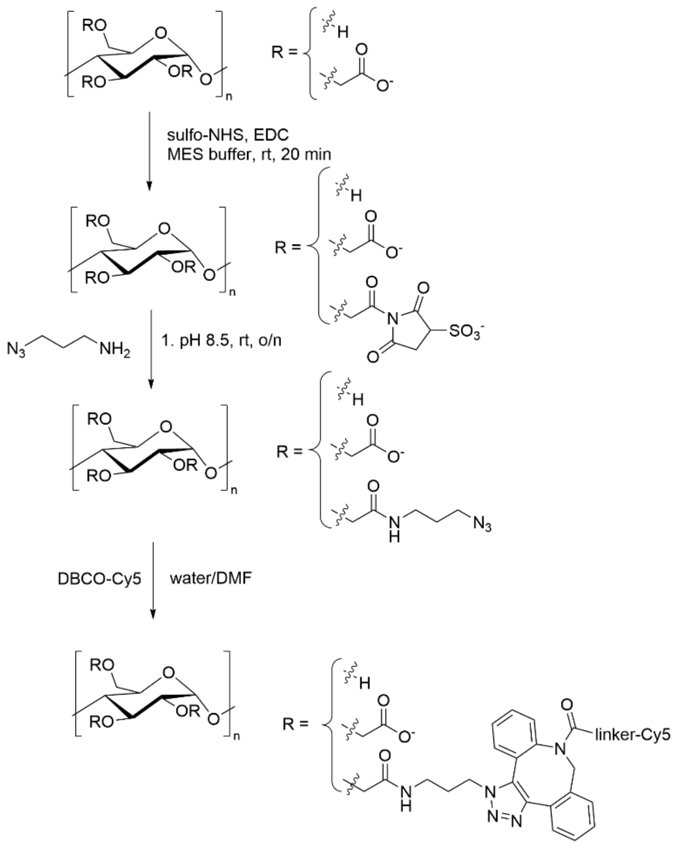

Supplementary Scheme 2. Synthetic route for Cy5-labeled Cm-Am.

Synthesis of poly(ethylene glycol)-poly(caprolactone-gradient-trimethylene carbonate)-polyglutamic acid (PEG-b-PCLgPTMC-b-PGA) terpolymer

The terpolymer (PEG-b-PCLgPTMC-b-PGA) was synthesized as described by a previously published procedure.[1] Step 1: Preparation of poly(ethylene glycol)-poly(caprolactone-gradient-trimethylene carbonate) (PEG-PCLgTMC). The organocatalyzed ring-opening polymerization of ε-caprolactone and trimethylene carbonate was performed, aiming for a composition of PEG_{44}-PCL_{50}-g-TMC_{50}. Monomethoxy-PEG-OH macroinitiator (2 kDa, 0.5 mmol) was weighed into an oven-dried round bottom flask and dried at 140 ºC under vacuum. After cooling down, distilled ε-caprolactone (ε-CL, 25 mmol) and recrystallized trimethylene carbonate (TMC, 25 mmol) were added under argon and dissolved in dry DCM (12 mL). The reaction was initiated by the addition of methanesulfonic acid (0.25 mmol ≈ 125 μL). The reaction mixture was stirred at 25 °C in a water bath and reaction progress was regularly checked. After completion (4-6 h) the reaction was quenched with DIPEA (1.5 mL), and the polymer was precipitated into ice cold methanol and lyophilized. This yielded 6.7 g of a waxy solid (67% yield). GPC analysis (using a PL gel 5 μm mixed D column, with THF as the solvent and calibration using polystyrene standards) yielded a polydispersity of 1.2.

Step 2: Chain-end modification with Boc-L-phenylalanine and deprotection. PEG-PCLgTMC (49 μmol) was dissolved in 5 mL acetonitrile. Then DMAP (25 μmol) and Boc-L-phenylalanine (0.25 mmol) were added and the solution was cooled to 0 ºC. After that, DCC (1.2 mmol) dissolved in ACN (1 mL) was added dropwise to the mixture. The reaction was stirred for 24 h at RT. After reaction completion the flask was placed in the freezer for 1 h and filtered through a plug of celite. The filtrate was concentrated and precipitated in cold Et2O (50 mL) to obtain the pure product. The resulting copolymer was then dissolved in DCM (5 mL), to which trifluoro acetic acid (5 mL) was added (on ice). The mixture was allowed to warm to RT and stirred for 2 h. After 2 h the solvent was evaporated and the copolymer was washed with $NaHCO_3$, 1 M NaCl and brine. Then it was dried over $MgSO_4$, filtered, concentrated, and finally precipitated from ice cold Et_2O. ^1H NMR spectroscopy showed that the signal arising from the terminal TMC group had disappeared, due to addition of phenylalanine at the terminus, and aromatic protons were visible at around 7.2-7.3 ppm. GPC analysis before and after deprotection yielded polydispersities of 1.1, indicating that TFA treatment did not facilitate copolymer hydrolysis.

Step 3: Polymerization and deprotection of N-carboxyanhydride γ-benzyl L-glutamate (BLG-NCA). Phe-terminated copolymer (57 μmol) was weighed into a Schlenk flask and dissolved in dry DMF (3 mL). Then NCA-BLG (5.7 mmol) was added under argon and the reaction was left under a constant flow of N_2 for 24 hrs. The product was precipitated into cold methanol and analyzed by ^1H NMR spectroscopy to confirm the overall composition and, in particular, the presence of benzylic and aromatic protons at 5.0-5.2 and 7.1-7.4 ppm, respectively. The resulting polymer (200 mg) was dissolved in THF (16 mL) and EtOH (2.5 mL) was added. The solution was degassed by bubbling N_2 through the solution for 20 min. Pd/C (10 mg) was added, the flask was filled with H_2 and the solution was left stirring overnight. After completion, the solution was filtered over celite. The filtrate was concentrated, precipitated into ice cold methanol, and lyophilized from dioxane. A colorless waxy solid was obtained. ^1H NMR spectroscopy was used to confirm successful deprotection of the PBLG units. The final composition of the terpolymer was $PEG_{44}P(CL_{63}gTMC_{52})PGA_7$. The composition of the resulting copolymer was confirmed by ^1H NMR spectroscopy in Supplementary Data 1,

comparing the protons of PEG (3.65 - 3.7 ppm), terminal methyl unit (singlet at 3.40 ppm) to PCL CH_2 (multiplet at 2.40 - 2.25 ppm) and PTMC CH_2 (multiplet at 2.2 - 1.8 ppm).

Supplementary Table 1. Overview of 14-3-3-binding peptides used in this work. Ahx = aminohexanoic acid, O1pen = (2-aminoethoxy)acetic acid, Ac = acetyl, p = phosphate.

Peptide name	Amino acid sequence with modifications	Binding mode
c-Raf pS233/pS259	FITC-Ahx-QHRY(pS)TPHAFTFNTSSPSSEGSLSQRQRST(pS)TPNVH-Ac	Bivalent
c-Raf S233/S259	FITC-Ahx-QHRYSTPHAFTFNTSSPSSEGSLSQRQRSTSTPNVH-Ac	No binding
BiExoS L423A	FITC-O1pen-QGLADALDLASGGGGGGGGGGQGLADALDLAS-Ac	Bivalent

Supplementary Table 2. Amino acid sequences and physicochemical parameters of the proteins used in this work. Strep-tags are shown in blue, His-tags are shown in red, TEV protease sites are shown in orange, and 14-3-3-binding domains are shown in green. Underlined residues indicate residues that can be phosphorylated. Physicochemical parameters were calculated using the online ProtParam tool (ExPASy). The extinction coefficient (ε) is given with all Cys residues reduced. Coincidentally, 14-3-3-His and GFP-c-Raf S233/S259 have the same extinction coefficient. [a]The protein does not contain any Trp residues and therefore the calculated extinction coefficient is not accurate. The concentration of this protein was determined by BCA assay. [b]Mass without phosphorylated residues.

Protein name	Amino acid sequence	Theoretical PI	M_w (kDa)	ε (M^{-1} cm^{-1}) at 280 nm
T14-3-3-cΔc	MAHHHHHHSSGLEVLFQGMAVAPTAREENVYMAKLA<br>EQAERYEEMVEFMEKVSNSLGSEELTVEERNLLSVAYK<br>NVIGARRASWRIISSIEQKEESRGNEEHVNSIREYRSKIEN<br>ELSKICDGILKLLDAKLIPSAASGDSKVFYLKMKGDYHR<br>YLAEFKTGAERKEAAESTLTAYKAAQDIATTELAPTHPI<br>RLGLALNFSVFYYEILNSPDRACNLAKQAFDEAIAELDT<br>LGEESYKDSTLIMQLLRDNLTLWTSD*	5.29	29.2	27390
His_6-SUMO-c-Raf S233/S259	MGSSHHHHHHLVPRGSGGSGILSDSEVNQEAKPEVKPE<br>VKPETHINLKVSDGSSEIFFKIKKTTPLRRLMEAFAKRQG<br>KEMDSLRFLYDGIRIQADQTPEDLDMEDNDIIEAHREQI<br>GGGTGGSQHRYSTPHAFTFNTSSPSSEGSLSQRQRSTSTP<br>NVH*	6.40	17.8	2980[a]
His_{10}-SUMO-c-Raf S233/S259	MGSHHHHHHHHHHSGGILSDSEVNQEAKPEVKPEVKPE<br>THINLKVSDGSSEIFFKIKKTTPLRRLMEAFAKRQGKEM<br>DSLRFLYDGIRIQADQTPEDLDMEDNDIIEAHREQIGGGT<br>GGSQHRYSTPHAFTFNTSSPSSEGSLSQRQRSTSTPNVH*	6.41	17.6	2980[a]
$(His_6)_2$-SUMO-c-Raf S233/S259	MGSSHHHHHHSGGSGGHHHHHHSGGILSDSEVNQEAKP<br>EVKPEVKPETHINLKVSDGSSEIFFKIKKTTPLRRLMEAF<br>AKRQGKEMDSLRFLYDGIRIQADQTPEDLDMEDNDIIEA<br>HREQIGGGTGGSQHRYSTPHAFTFNTSSPSSEGSLSQRQR<br>STSTPNVH*	6.48	18.3	2980[a]
PKA-His	MGHHHHHHSSGENLYFQGSVKEFLAKAKEDFLKKWES<br>PAQNTAHLDQFERIKTLGTGSFGRVMLVKHKETGNHYA<br>MKILDKQKVVKLKQIEHTLNEKRILQAVNFPFLVKLEFS<br>FKDNSNLYMVMEYVPGGEMFSHLRRIGRFSEPHARFYA<br>AQIVLTFEYLHSLDLIYRDLKPENLLIDQQGYIQVTDFGF<br>AKRVKGRTWTLCGTPEYLAPEIILSKGYNKAVDWWAL<br>GVLIYEMAAGYPPFFADQPIQIYEKIVSGKVRFPSHFSSD<br>LKDLLRNLLQVDLTKRFGNLKNGVNDIKNHKWFATTD<br>WIAIYQRKVEAPFIPKFKGPGDTSNFDDYEEEEIRVSINE<br>KCGKEFSEFSSGSSGWSHPQFEK*	8.71	42.8[b]	60850
GFP-BiExoS L423A	MGGSVKMGASKGEELFTGVVPILVELDGDVNGHKFSVR<br>GEGEGDATNGKLTLKFICTTGKLPVPWPTLVTTLTYGV<br>QCFSRYPDHMKQHDFFKSAMPEGYVQERTISFKDDGTY<br>KTRAEVKFEGDTLVNRIELKGIDFKEDGNILGHKLEYNF<br>NSHNVYITADKQKNGIKANFKIRHNVEDGSVQLADHYQ<br>QNTPIGDGPVLLPDNHYLSTQSALSKDPNEKRDHMVLL<br>EFVTAAGITHGMDELYKTENLYFQGGSGTGGSQGLADA<br>LDLASGGSGSGTGGGQGLADALDLASGGSGGSGGSWS<br>HPQFEK*	5.45	33.3	25900

Supplementary Figures

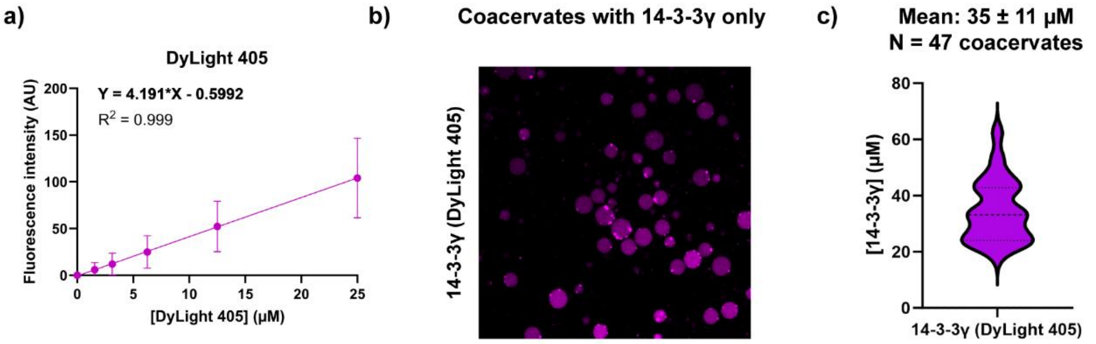

Supplementary Figure 1. Measurement of the local concentration of 14-3-3 in the coacervates. a) Calibration curve of free DyLight 405 NHS ester in coacervate buffer, using the same settings used for the coacervate samples. b) Confocal micrograph of coacervates loaded with DyLight 405-labeled 14-3-3γ (100 nM). c) Quantification of micrograph in panel b, revealing the distribution of local concentrations of 14-3-3γ in the coacervates after correcting for the degree of labeling of the protein, which was 0.57.

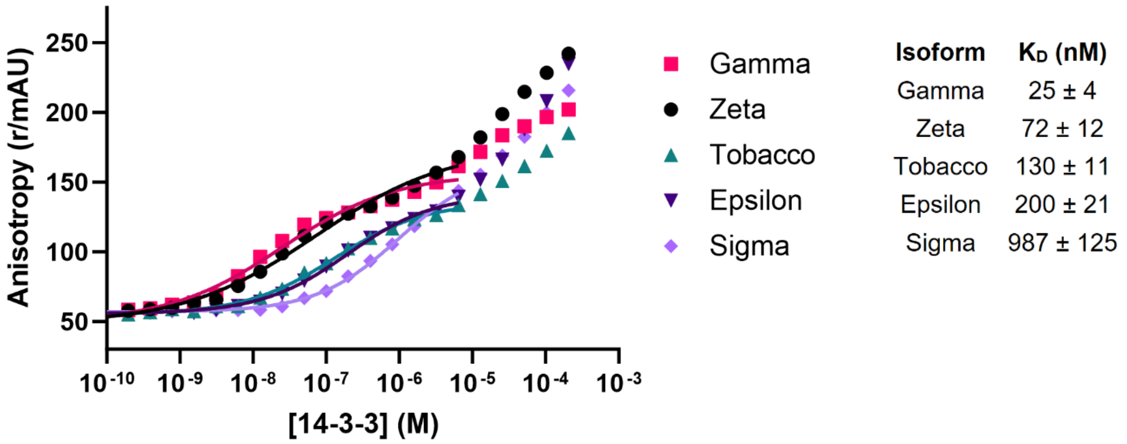

Supplementary Figure 2. Protein titration of different 14-3-3 isoforms to the FITC-c-Raf pS233/pS259 peptide, as determined by fluorescence anisotropy assay. Symbols represent the mean of a triplicate measurement, with the error bar obscured by the symbols. Lines show fits by a 4-parameter logistic model, fitted until the 2 µM data point. At higher concentrations, a second binding event was observed and therefore this second binding event was not fitted. The table shows the resulting K_D obtained from the fit, shown as mean with the standard deviation.

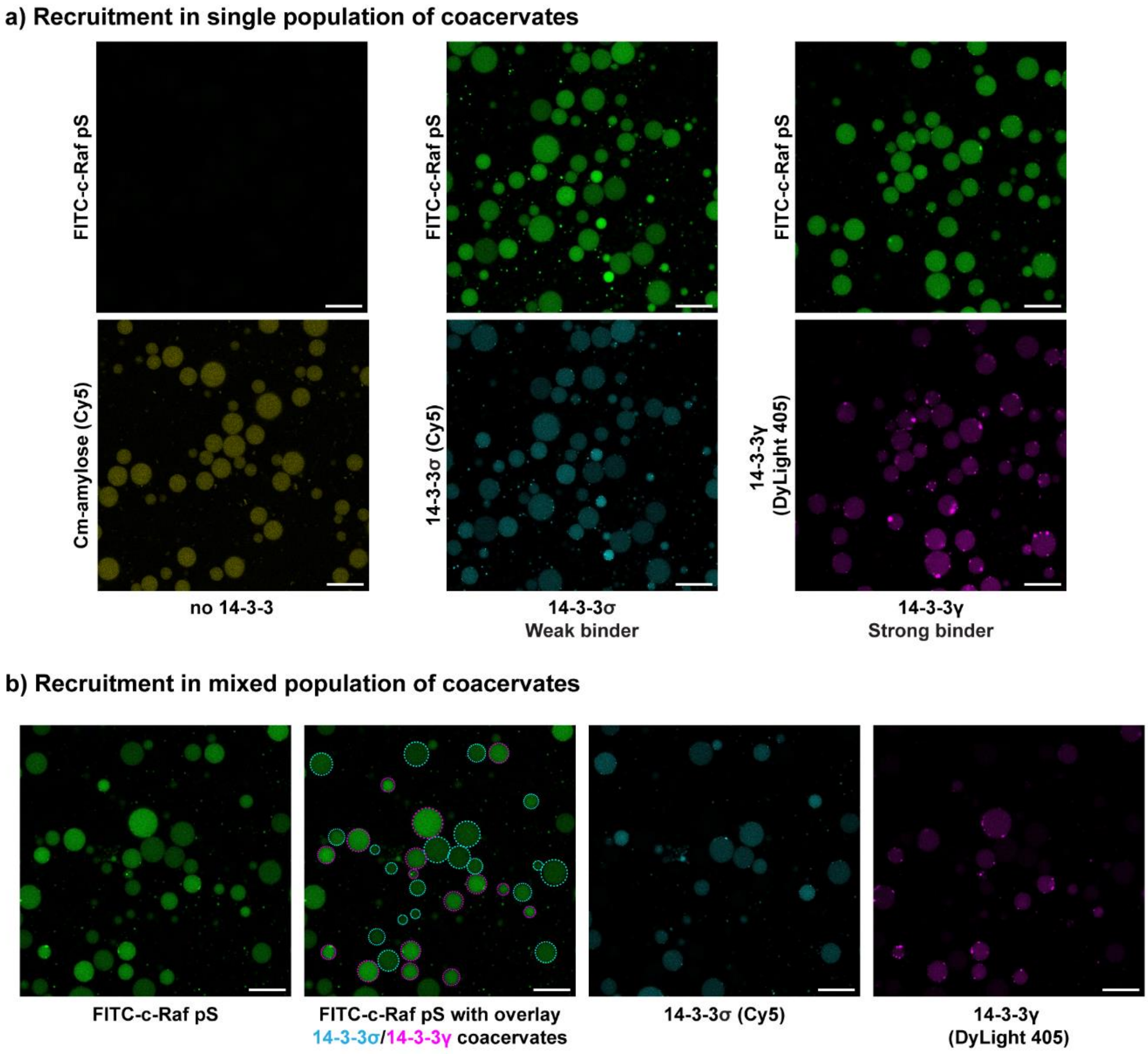

Supplementary Figure 3. Uncropped images related to Figure 2. a) Confocal micrographs of FITC-c-Raf pS (25 nM) in the absence or presence of either 14-3-3γ or 14-3-3σ (100 nM) after overnight equilibration in a single population of coacervates. The sample without 14-3-3 was supplied with Cy5-labeled Cm-amylose as imaging agent. Scale bar: 25 µm. b) Confocal micrographs of the directed uptake of FITC-c-Raf pS (25 nM) in a mixed population of coacervates loaded with 14-3-3γ or 14-3-3σ (100 nM) after overnight equilibration. Scale bar: 25 µm.

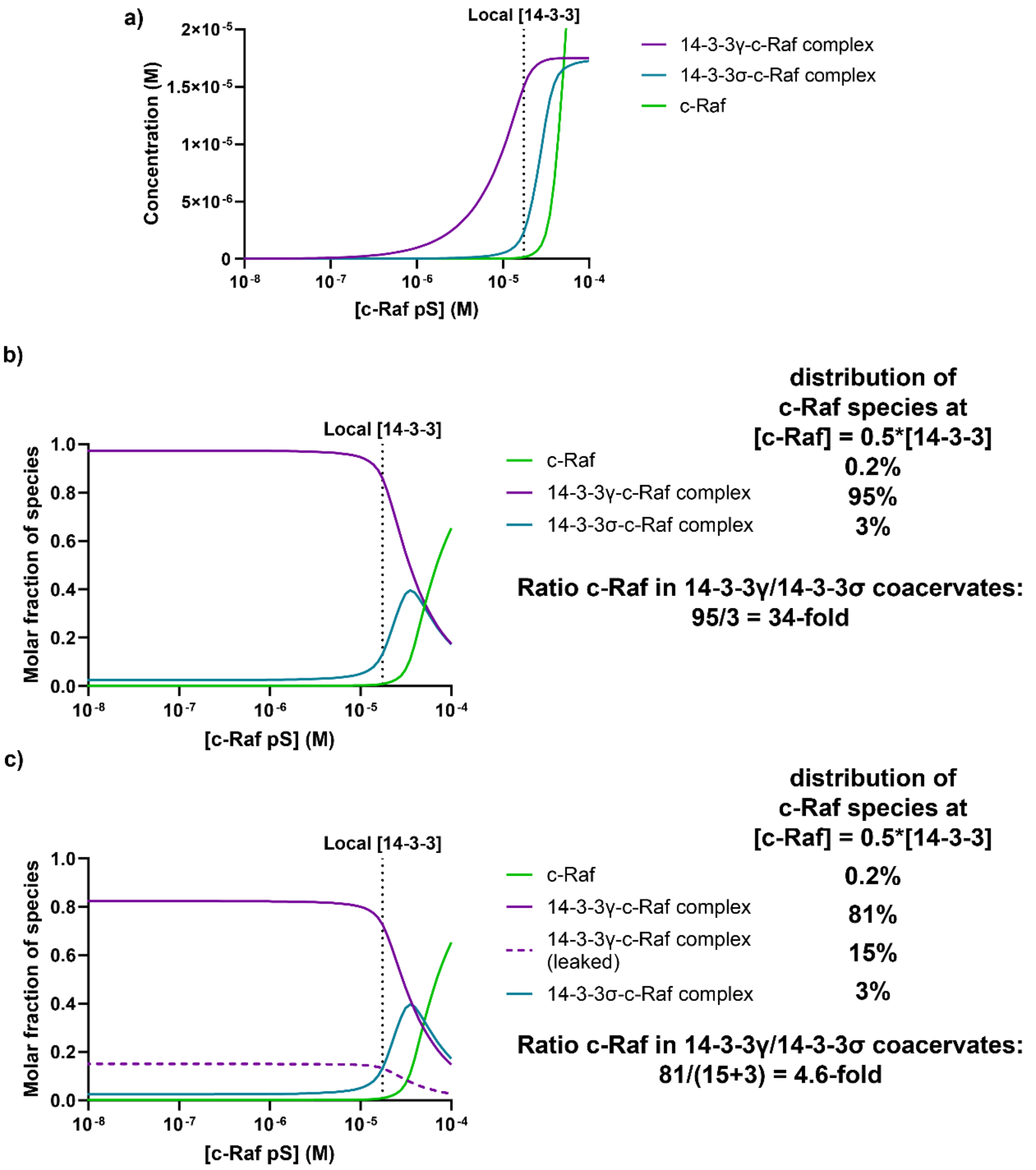

Supplementary Figure 4. a) Local concentration of species at increasing concentration of c-Raf phosphopeptide (c-Raf pS) in a system with 17.5 μM of 14-3-3σ and 14-3-3γ dimers (1:1 binding), as calculated by a thermodynamic equilibrium model. The concentration is based on the quantification of 14-3-3 monomer concentration in the coacervates using fluorescence calibration curve. b) Transformation of the data into molar fractions of complexes of c-Raf pS, and the resulting difference in c-Raf pS uptake between the coacervates with both isoforms. c) Molar fractions of complexes of c-Raf pS, taking into account the 14-3-3γ that has leaked into the 14-3-3σ coacervates (dashed line). Also shown is the calculation of the enrichment of the c-Raf pS peptide into the 14-3-3γ coacervates. The occupancy was determined via interpolation of model-generated data using a four-parameter logistic model (GraphPad 10.2.1).

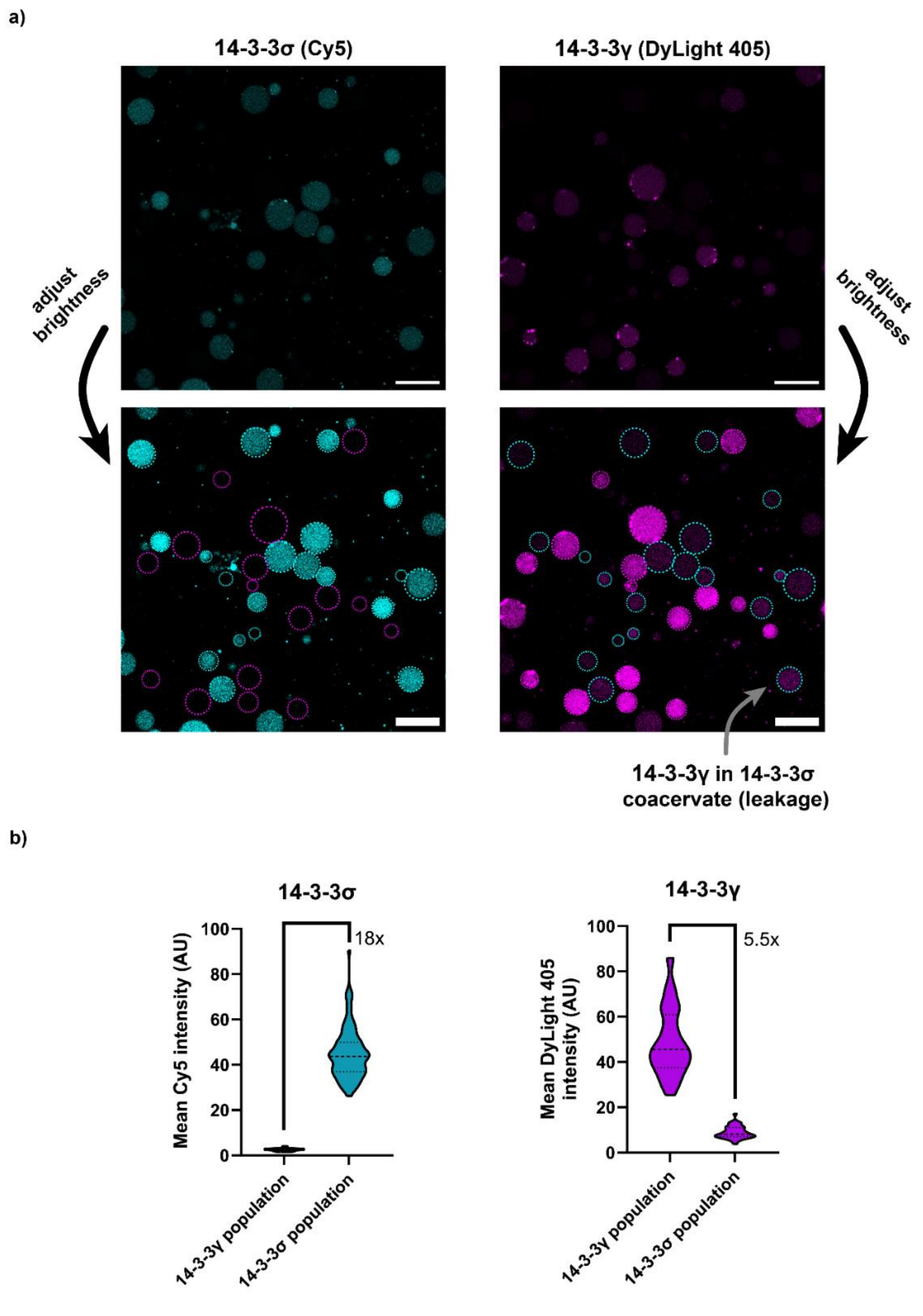

Supplementary Figure 5. Uncropped images and quantification of 14-3-3 signal related to Figure 2. a) Confocal micrographs of a 1:1 (v/v) mixed sample of coacervates containing 14-3-3γ or 14-3-3σ (100 nM) after overnight equilibration. The brightness of the bottom row of images was adjusted for visibility, and cyan and magenta outlines were added as a visual guide. Scale bar: 25 µm. b) Quantifications of the 14-3-3 isoforms in both coacervate populations from the unedited image.

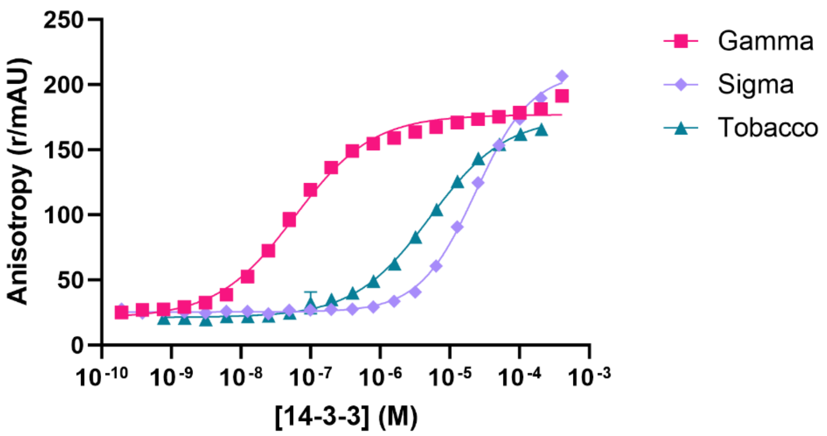

Isoform	K_D (μM)
Gamma	0.059 ± 0.004
Tobacco	5.2 ± 0.2
Sigma	23 ± 1

Supplementary Figure 6. Protein titration of different 14-3-3 isoforms to the FITC-BiExoS L423A peptide, as determined by fluorescence anisotropy assay. Symbols represent the mean of a triplicate measurement, with the error bar obscured by the symbols. Lines show fits by a 4-parameter logistic model. The table shows the resulting K_D obtained from the fit, shown as mean with the standard deviation.

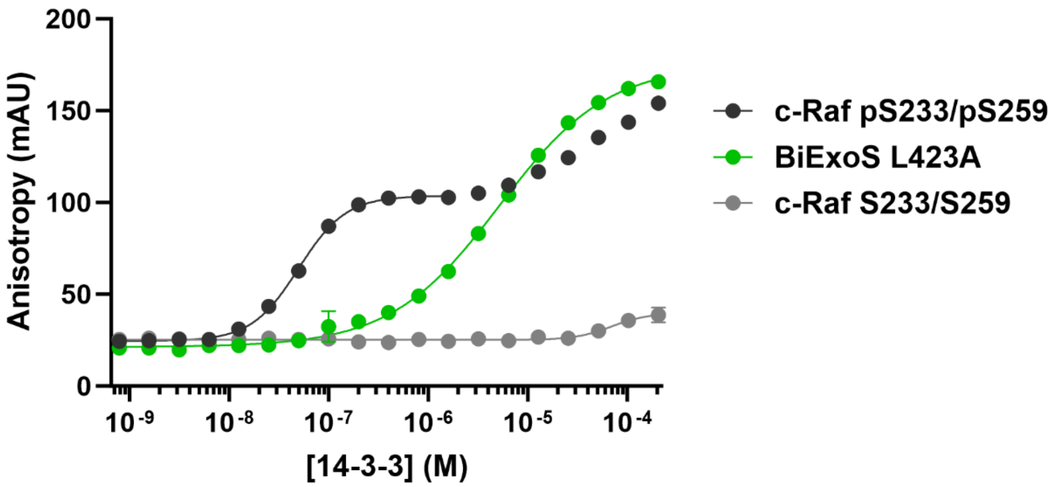

Supplementary Figure 7. Protein titration of T14-3-3 to different peptides, as determined by fluorescence anisotropy assay. Symbols represent the mean of a triplicate measurement, with the error bar obscured by the symbols. Lines show fits by a 4-parameter logistic model, fitted until the 2 μM data point in the case of the c-Raf pS233/pS259 peptide. At higher concentrations, a second binding event was observed for this peptide, which was not fitted.

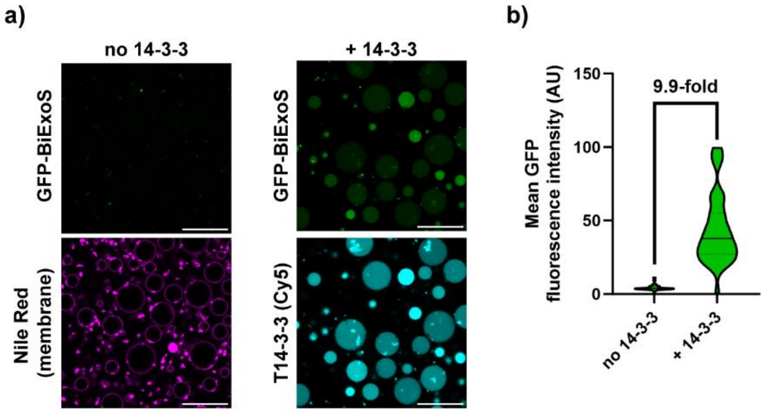

Supplementary Figure 8. GFP-BiExoS is taken up in coacervates in a 14-3-3-dependent manner. a) Confocal micrographs of GFP-BiExoS (100 nM) in the absence or presence of T14-3-3 (100 nM) after overnight equilibration. The sample without 14-3-3 was supplied with Nile Red in the terpolymer stock solution to a final concentration 450 nM for imaging purposes. Scale bar: 25 µm. b) Quantification of the micrographs in panel a using the uncropped images (not shown). The fold change relates to the mean of the populations.

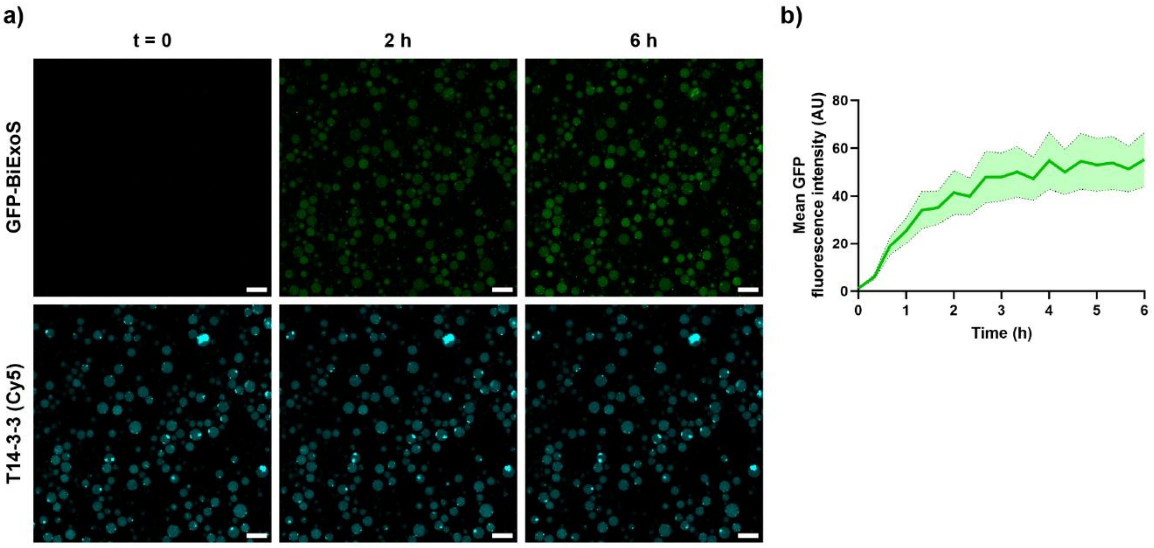

Supplementary Figure 9. GFP-BiExoS is taken up in coacervates within 5 hours. a) Confocal micrographs of coacervates loaded with 100 nM of Cy5-labeled T14-3-3, to which 100 nM of GFP-BiExoS was added directly before the initial timepoint and followed over time. Scale bar: 25 µm. b) Quantification of the GFP-BiExoS signal from the same measurement as panel a.

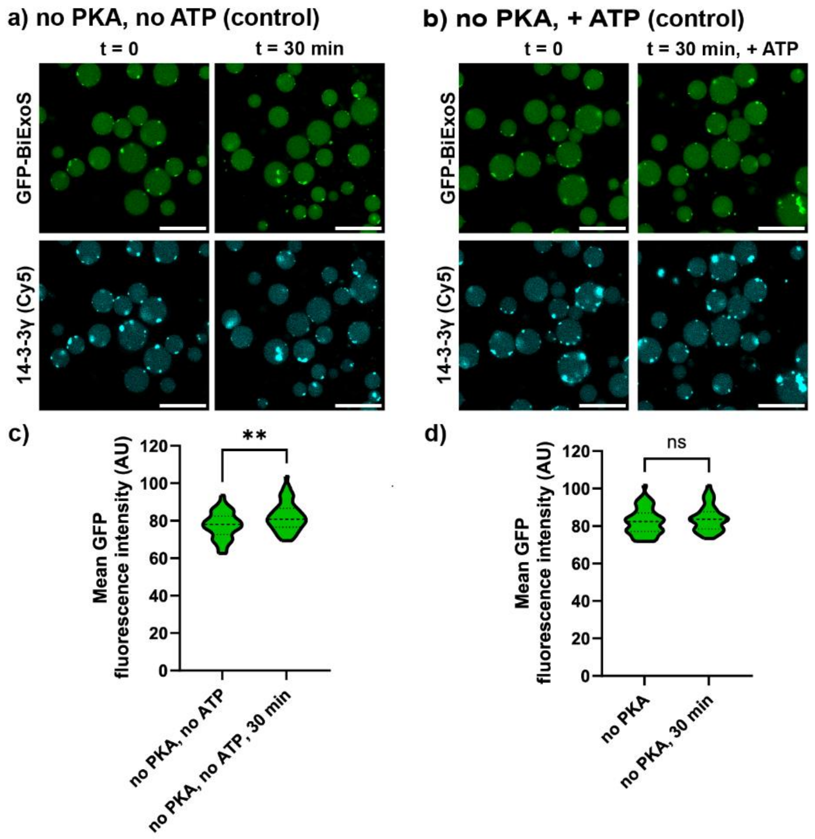

Supplementary Figure 10. a, b) Control samples for the displacement of GFP-BiExoS by His_{10}-SUMO-c-Raf in the absence of kinase PKA, in the absence (a) or presence (b) of ATP. In both samples, no release of GFP-BiExoS over 30 min was observed. Conditions: 100 nM of 14-3-3γ (Cy5-labeled), 50 nM of His_{10}-SUMO-c-Raf, 50 nM of GFP-BiExoS, bulk concentrations. Scale bar: 25 µm. c, d) Quantification of the GFP-BiExoS signal in micrographs in panels a and b. Statistical differences were analyzed by unpaired t-test, with $N \geq 49$ coacervates across 2 imaging positions in the same sample. Dashed lines represent the median and dotted lines represent the upper and lower quartiles. ns: no statistical difference, **: $p < 0.01$.

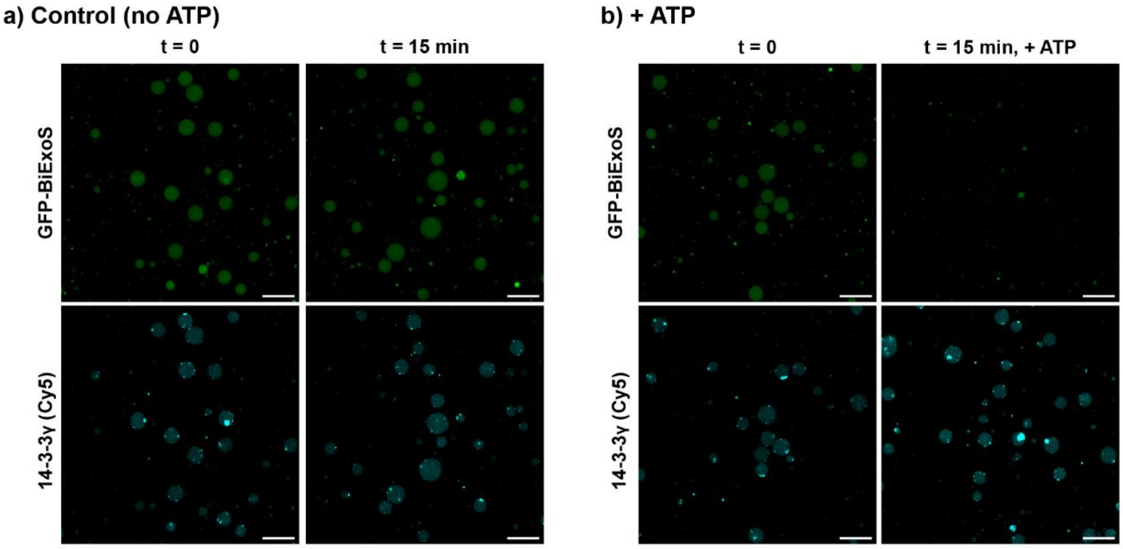

Supplementary Figure 11. Uncropped micrographs showing the displacement of GFP-BiExoS by His_{10}-SUMO-c-Raf from coacervates after phosphorylation of the c-Raf domain. Confocal micrographs of the control sample in the absence of ATP (a) and the sample demonstrating triggered release of GFP-BiExoS in the presence of ATP (b). Conditions: 100 nM of 14-3-3γ (Cy5-labeled), 10 nM of PKA (His-tagged), 50 nM of His_{10}-SUMO-c-Raf, 50 nM of GFP-BiExoS, bulk concentrations. Scale bar: 25 μm.

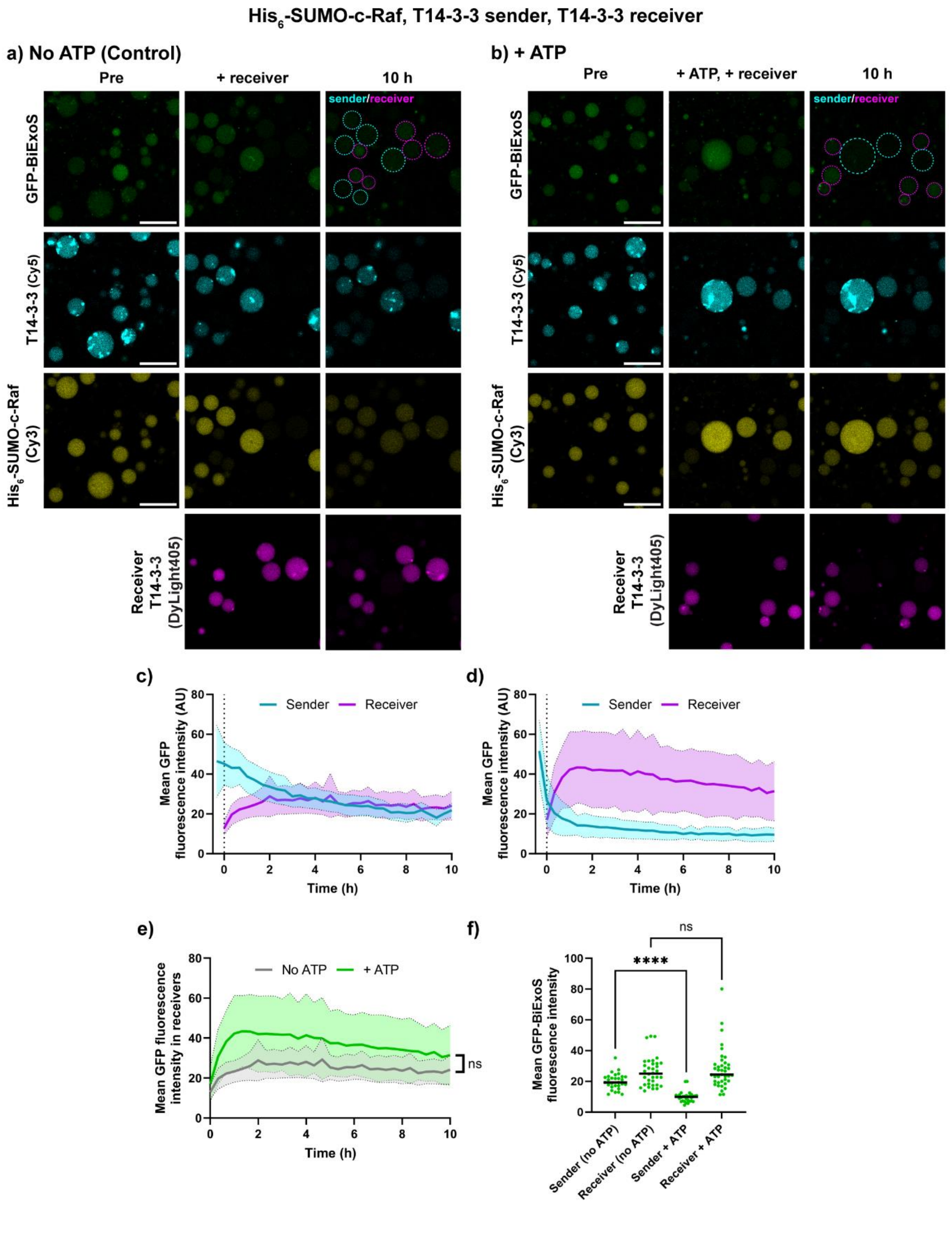

Supplementary Figure 12. Confocal micrographs of GFP-BiExoS shuttling from T14-3-3-loaded sender coacervates to T14-3-3-loaded receiver coacervates induced by the enzymatic phosphorylation of co-assembled His$_6$-SUMO-c-Raf (loaded in the sender coacervates). a, b) Confocal micrographs of the control sample in the absence of ATP (a) and in the presence of ATP (b). Conditions: Senders were loaded with 100 nM of T14-3-3 (Cy5-labeled), 10 nM of PKA (His-tagged), 50 nM of His$_6$-SUMO-c-Raf, and 50 nM of GFP-BiExoS. Receiver coacervates were loaded with 100 nM of T14-3-3 (DyLight 405-labeled). Colored outlines of sender and receiver coacervates were added as a visual guide. Scale bar: 25 µm. c, d) Quantification of the GFP-BiExoS signal in micrographs over time in the samples of

panels a and b, respectively. e, f) Quantification of micrographs over time comparing the receiver populations (e) and both populations at distinct positions at the 10 h timepoint (f), showing the GFP signal in the sender and receiver populations of coacervates. Statistical differences were analyzed by Dunn's test with correction for multiple comparisons, with $N \geq 47$ coacervates across multiple imaging positions in the same sample. Dashed lines represent the median and dotted lines represent the upper and lower quartiles. Ns: no statistical differences, ****: $p < 0.0001$. Time traces are shown as mean ± standard deviation.

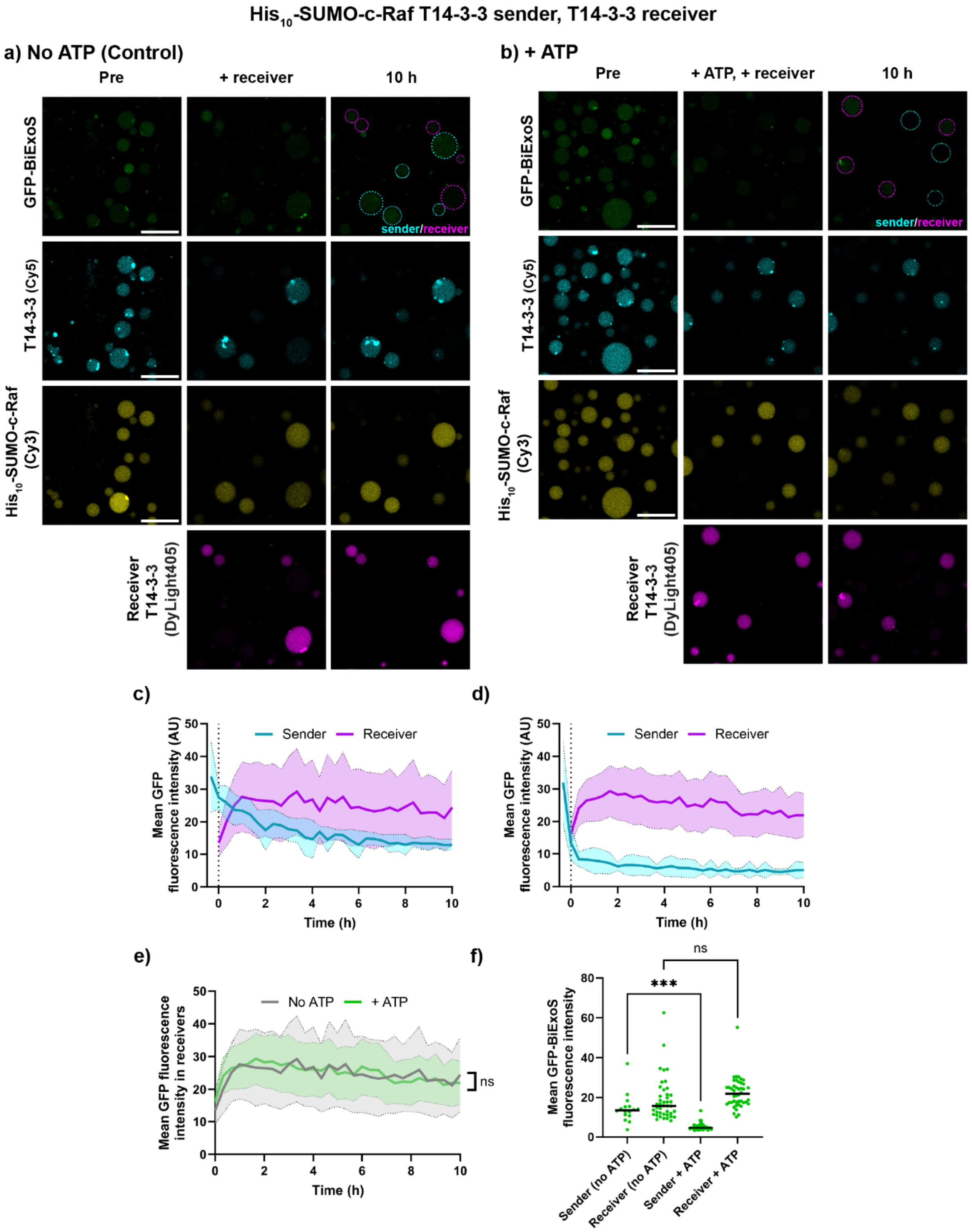

Supplementary Figure 13. Confocal micrographs of GFP-BiExoS shuttling from T14-3-3-loaded sender coacervates to T14-3-3-loaded receiver coacervates induced by the enzymatic phosphorylation of co-assembled His_{10}-SUMO-c-Raf (loaded in the sender coacervates). a, b) Confocal micrographs of the control sample in the absence of ATP (a) and in the presence of ATP (b). Conditions: Senders were loaded with 100 nM of T14-3-3 (Cy5-labeled), 10 nM of PKA (His-tagged), 50 nM of His_{10}-SUMO-c-Raf, and 50 nM of GFP-BiExoS. Receiver coacervates were loaded with 100 nM of T14-3-3 (DyLight 405-labeled). Colored outlines of sender and receiver coacervates were added as a visual guide. Scale bar: 25 µm. c, d) Quantification of the GFP-BiExoS signal in micrographs over time in the samples of

panels a and b, respectively. e, f) Quantification of micrographs over time comparing the receiver populations (e) and both populations at distinct positions at the 10 h timepoint (f), showing the GFP signal in the sender and receiver populations of coacervates. Statistical differences were analyzed by nonparametric Dunn's test with correction for multiple comparisons, with $N \geq 16$ coacervates across multiple imaging positions in the same sample. Dashed lines represent the median and dotted lines represent the upper and lower quartiles. Ns: no statistical differences, ****: $p < 0.0001$. Time traces are shown as mean ± standard deviation.

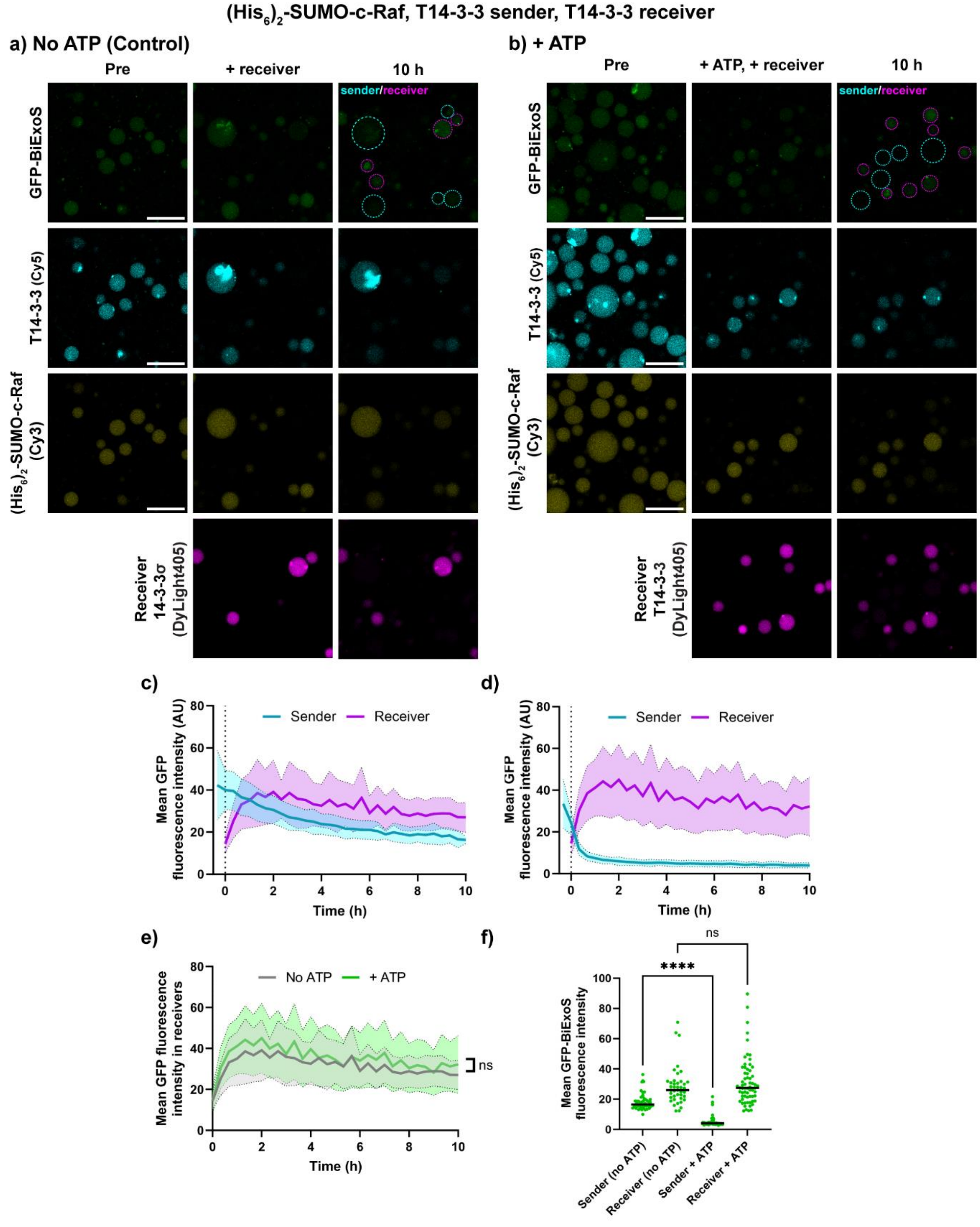

Supplementary Figure 14. Confocal micrographs of GFP-BiExoS shuttling from T14-3-3-loaded sender coacervates to T14-3-3-loaded receiver coacervates induced by the enzymatic phosphorylation of co-assembled $(His_6)_2$-SUMO-c-Raf (loaded in the sender coacervates). a, b) Confocal micrographs of the control sample in the absence of ATP (a) and in the presence of ATP (b). Conditions: Senders were loaded with 100 nM of T14-3-3 (Cy5-labeled), 10 nM of PKA (His-tagged), 50 nM of $(His_6)_2$-SUMO-c-Raf, and 50 nM of GFP-BiExoS. Receiver coacervates were loaded with 100 nM of T14-3-3 (DyLight 405-labeled). Colored outlines of sender and receiver coacervates were added as a visual guide. Scale bar: 25 µm. c, d) Quantification of the GFP-BiExoS signal in micrographs over time in the

samples of panels a and b, respectively. e, f) Quantification of micrographs over time comparing the receiver populations (e) and both populations at distinct positions at the 10 h timepoint (f), showing the GFP signal in the sender and receiver populations of coacervates. Statistical differences were analyzed by Dunn's test with correction for multiple comparisons, with N ≥ 29 coacervates across multiple imaging positions in the same sample. Dashed lines represent the median and dotted lines represent the upper and lower quartiles. Ns: no statistical differences, ****: $p < 0.0001$. Time traces are shown as mean ± standard deviation.

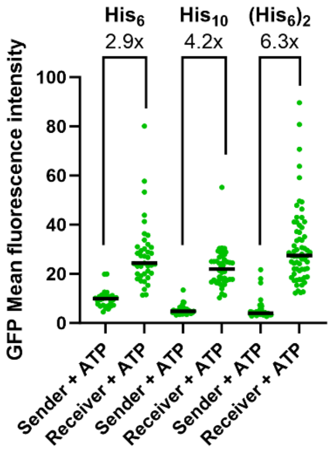

Supplementary Figure 15. Comparison between the GFP-BiExoS signal in sender and receiver populations of coacervates from the experiments of Supplementary Figs. 12-14. Higher His-tag affinity on His-SUMO-c-Raf was found to yield a greater difference in GFP-BiExoS signal between senders and receivers, both loaded with T14-3-3. The data shown is in the presence of ATP, for samples with different His-SUMO-c-Raf proteins, with the His-tag length shown above the data.

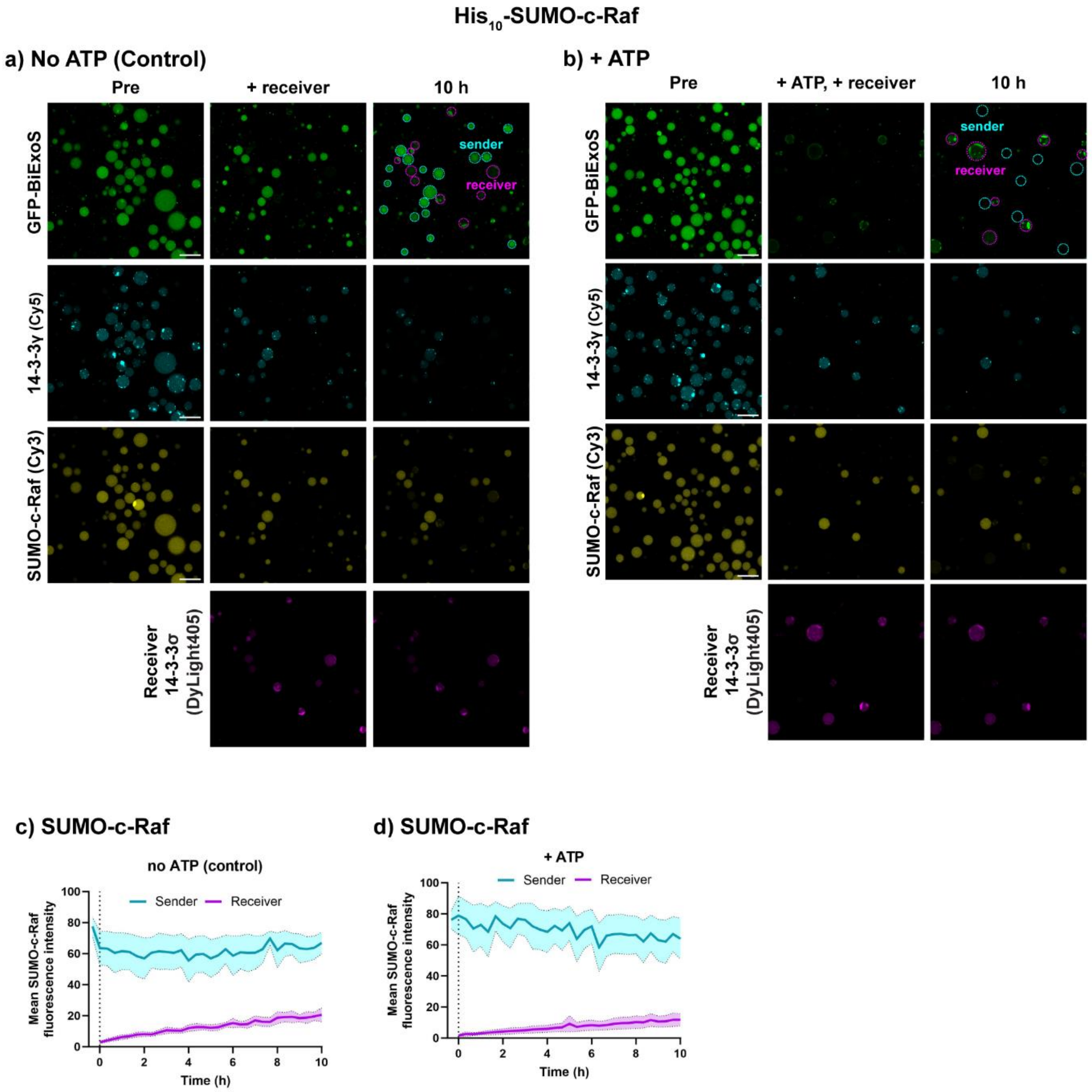

Supplementary Figure 16. Uncropped images and additional channels showing displacement of GFP-BiExoS from 14-3-3γ-loaded coacervates to 14-3-3σ-loaded coacervates in the presence of His_{10}-SUMO-c-Raf (loaded in the sender coacervates). a, b) Confocal micrographs of the control sample in the absence of ATP (a) and the sample demonstrating triggered signaling of GFP-BiExoS in the presence of ATP (b). Conditions: Senders were loaded with 100 nM of 14-3-3γ (Cy5-labeled), 10 nM of PKA (His-tagged), 50 nM of His_{10}-SUMO-c-Raf, 50 nM of GFP-BiExoS. Receiver coacervates were loaded with 500 nM of 14-3-3σ (DyLight 405-labeled). Colored outlines of sender and receiver coacervates were added as a visual guide. Scale bar: 25 µm. c, d) Quantification of the His_{10}-SUMO-c-Raf (Cy3-labeled) signal in micrographs over time in the samples of panels a and b, respectively.

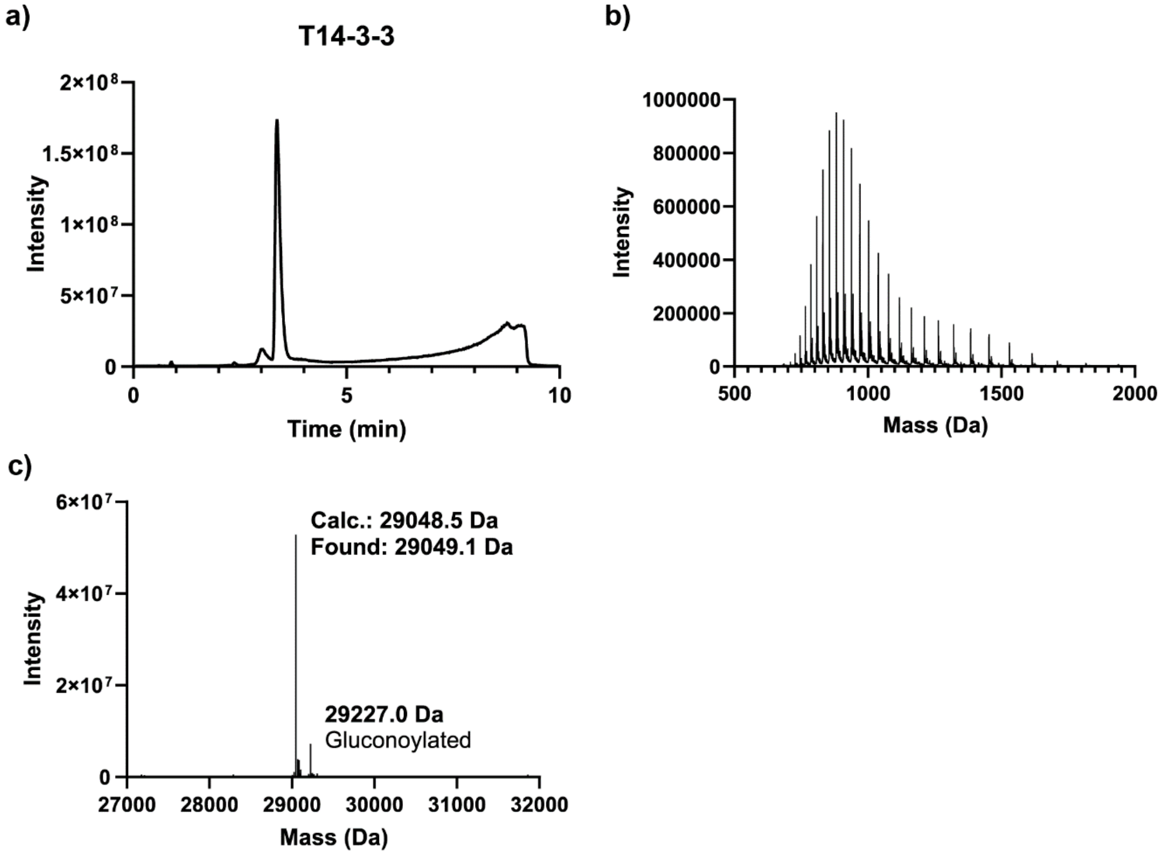

Supplementary Figure 17. a) LC-MS Q-ToF chromatogram, b) m/z spectrum, and c) deconvoluted mass of T14-3-3. Expected mass for T14-3-3: 29048.5 Da (after N-terminal Met excision). Found masses: 29049.1 Da and 29227.0 Da (gluconoylated).[4]

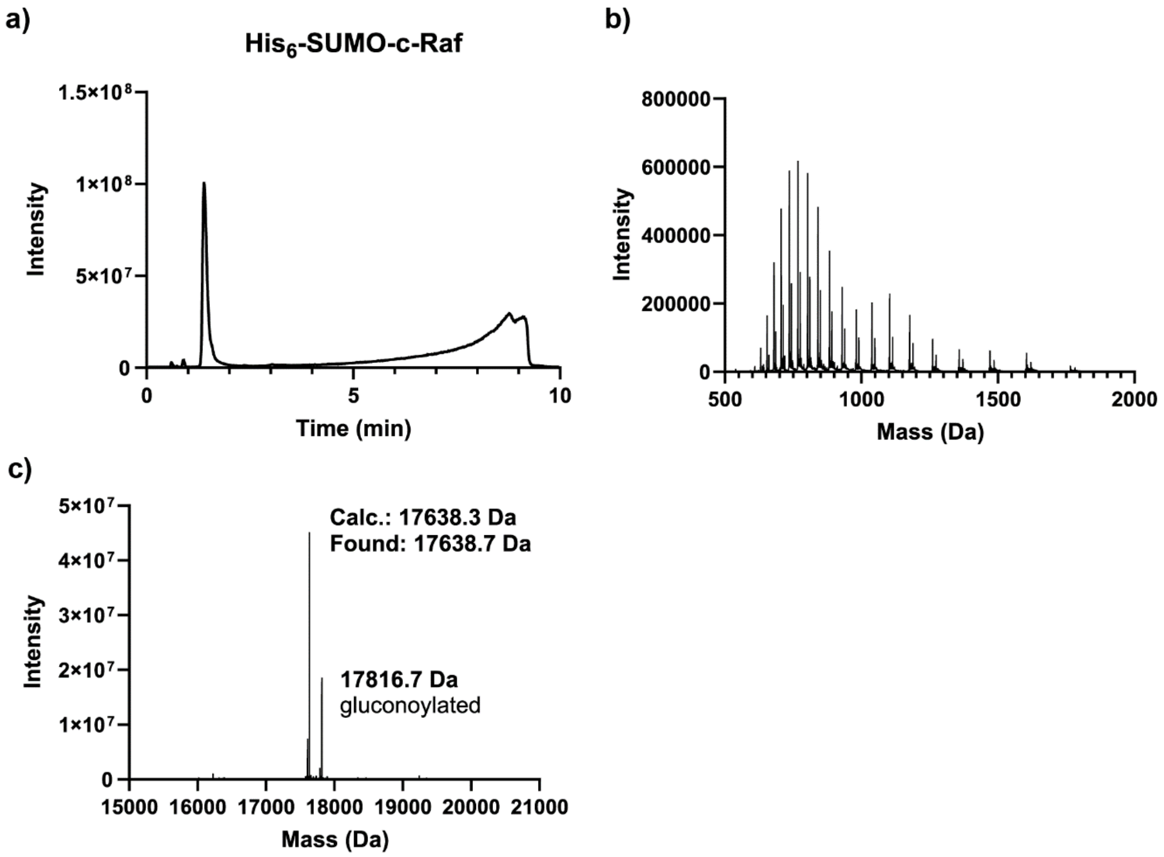

Supplementary Figure 18. a) LC-MS Q-ToF chromatogram, b) m/z spectrum, and c) deconvoluted mass of His_6-SUMO-c-Raf. Expected mass for His_6-SUMO-c-Raf: 17638.3 Da (after N-terminal Met excision). Found masses: 17638.7 Da and 17816.7 Da (gluconoylated).[4]

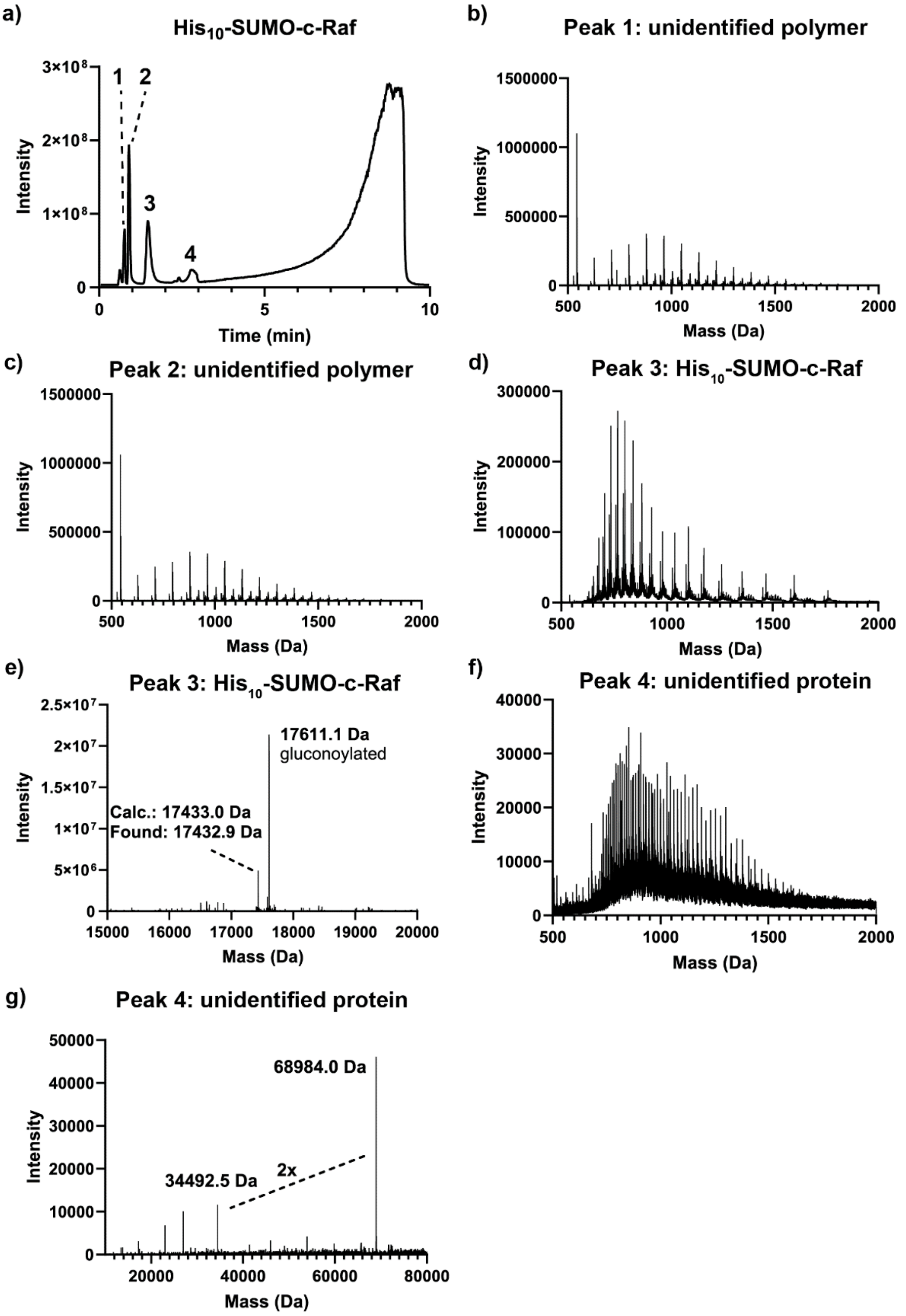

Supplementary Figure 19. a) LC-MS Q-ToF chromatogram of His_{10}-SUMO-c-Raf. b, c) m/z spectra for unidentified polymers found in peaks 1 and 2 of the chromatogram, respectively. d, e) m/z spectrum (d) and deconvoluted mass (e) of peak 3, corresponding to His_{10}-SUMO-c-Raf. Expected mass for His_{10}-SUMO-c-Raf: 17433.0 Da (after N-terminal Met excision). Found masses: 17432.9 Da and 17611.1 Da (gluconoylated).[4] f, g) m/z spectrum (f) and deconvoluted mass (g) of peak 4, corresponding to an unidentified protein with mass 68984.0 Da.

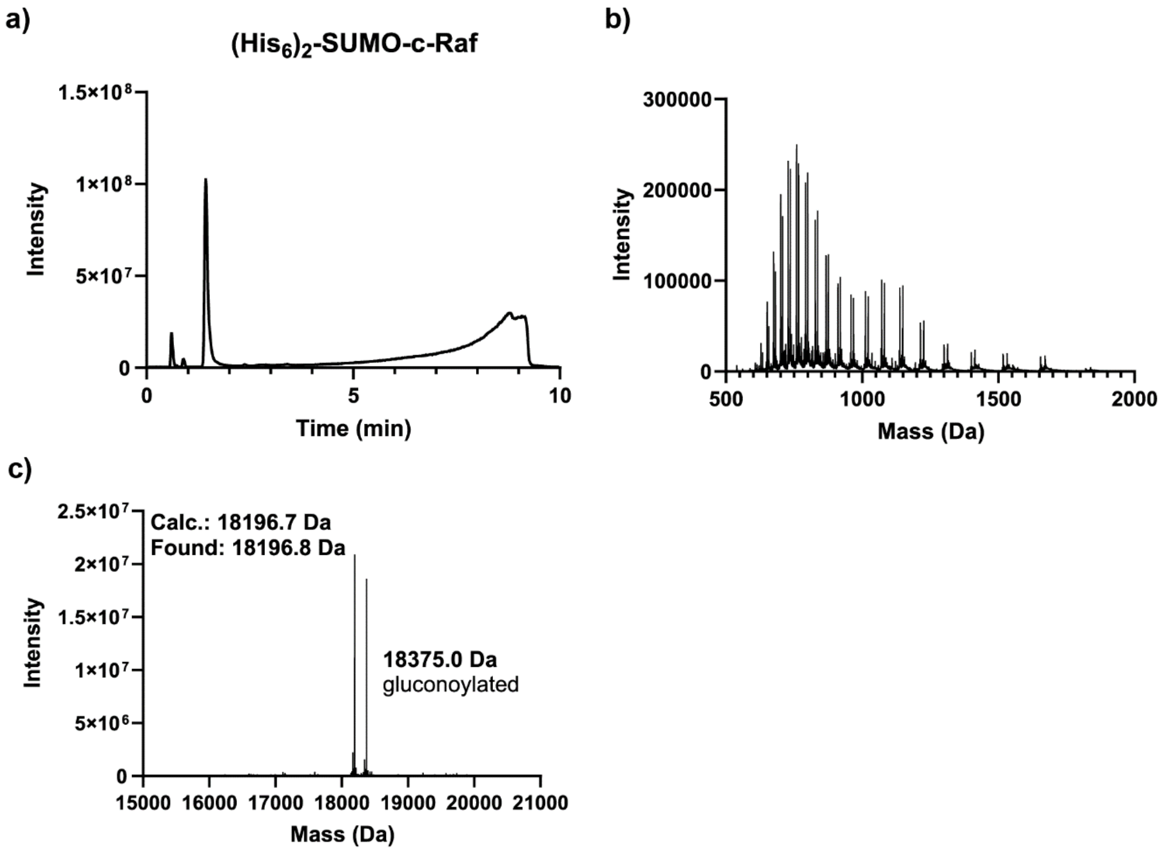

Supplementary Figure 20. a) LC-MS Q-ToF chromatogram, b) m/z spectrum, and c) deconvoluted mass of $(His_6)_2$-SUMO-c-Raf. Expected mass for $(His_6)_2$-SUMO-c-Raf: 18196.7 Da (after N-terminal Met excision). Found masses: 18196.8 Da and 18375.0 Da (gluconoylated).[4]

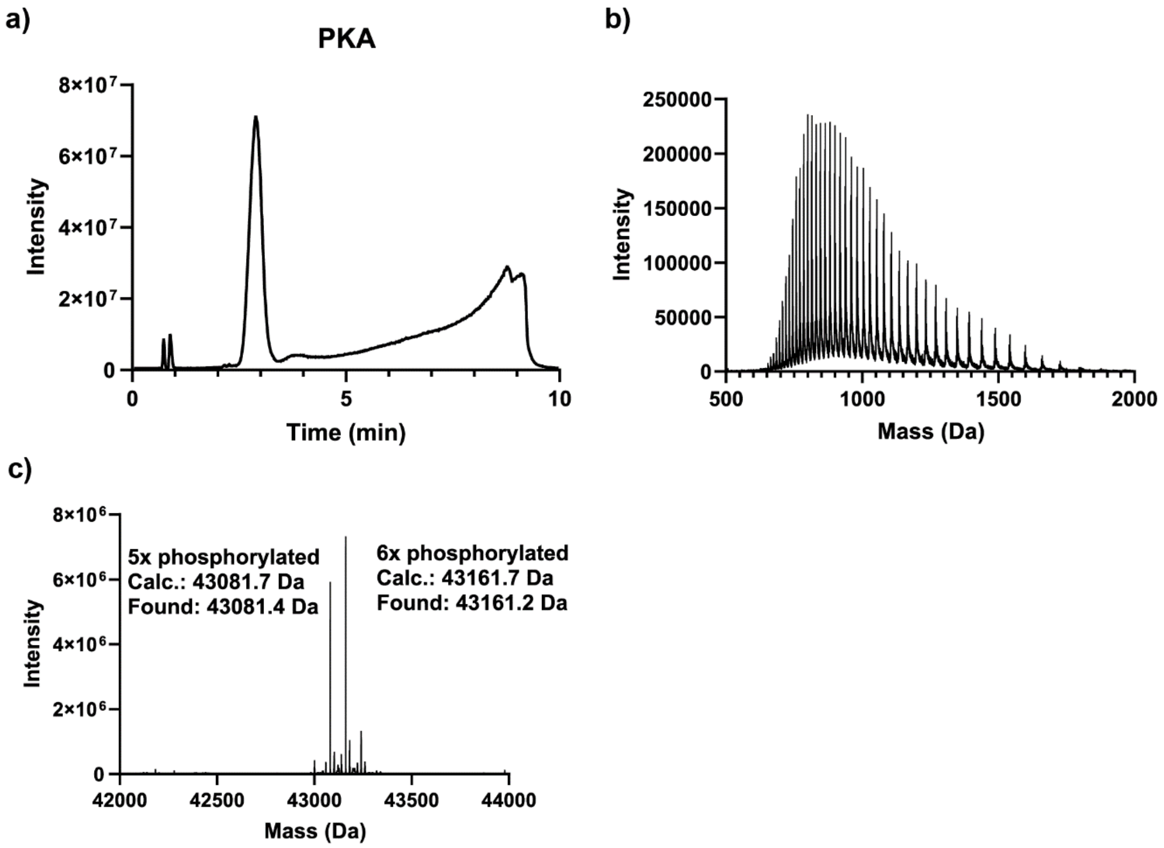

Supplementary Figure 21. a) LC-MS Q-ToF chromatogram, b) m/z spectrum, and c) deconvoluted mass of PKA. Expected mass for PKA: 43081.7 Da (after N-terminal Met excision and 5x autophosphorylation) and 43161.7 Da (after N-terminal Met excision and 6x autophosphorylation). Found masses: 43081.4 Da and 43161.2 Da.

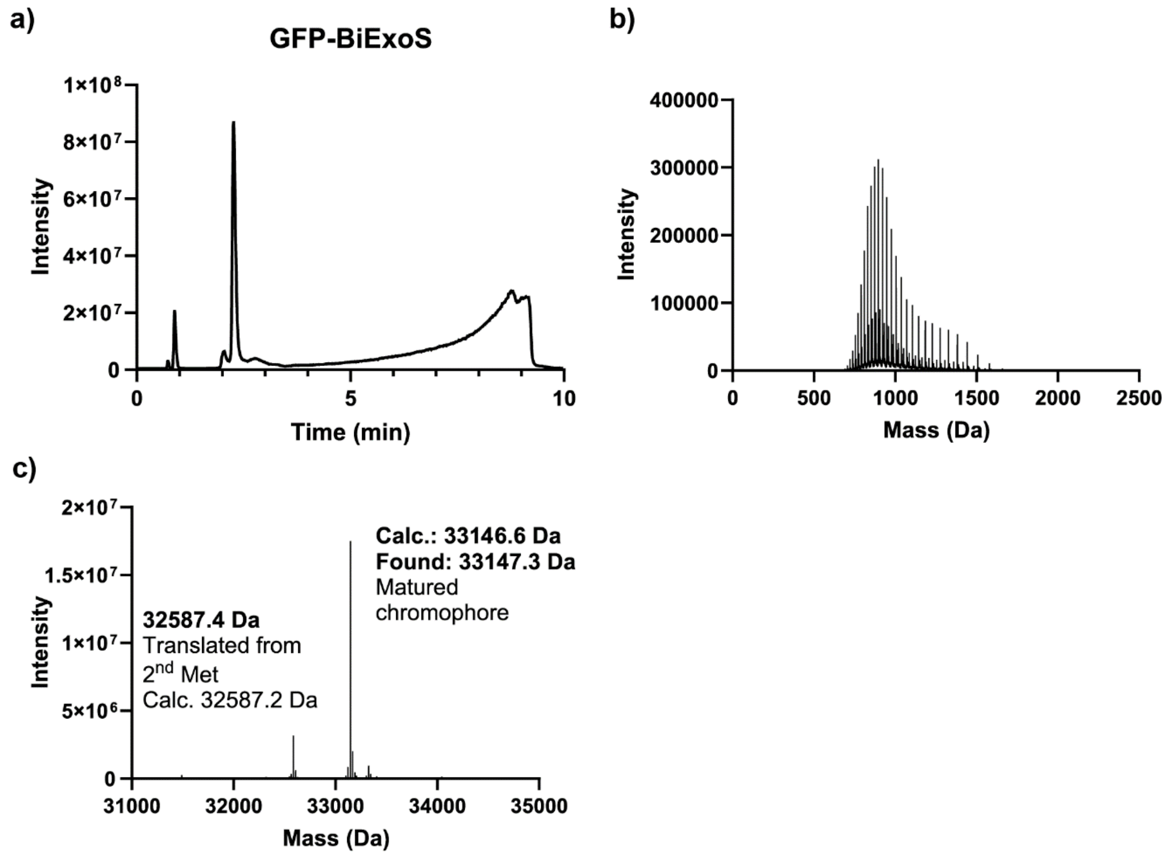

Supplementary Figure 22. a) LC-MS Q-ToF chromatogram, b) m/z spectrum, and c) deconvoluted mass of GFP-BiExoS. Expected mass for GFP-BiExoS: 33146.6 Da (after N-terminal Met excision and chromophore maturation with a loss of 20.0 Da). Found masses: 33147.3 Da (with matured chromophore) and a minor signal at 32587.4 Da (translated from Met in position 7).

Supplementary References

1. Mason, A. F., Buddingh, B. C., Williams, D. S. & van Hest, J. C. M. Hierarchical Self-Assembly of a Copolymer-Stabilized Coacervate Protocell. *J. Am. Chem. Soc.* **139**, 17309–17312 (2017).

2. Altenburg, W. J. *et al.* Programmed spatial organization of biomacromolecules into discrete, coacervate-based protocells. *Nat. Commun.* **11**, 6282 (2020).

3. Totaro, K. A. *et al.* Systematic Investigation of EDC/sNHS-Mediated Bioconjugation Reactions for Carboxylated Peptide Substrates. *Bioconjug. Chem.* **27**, 994–1004 (2016).

4. Geoghegan, K. F. *et al.* Spontaneous α-N-6-phosphogluconoylation of a 'His tag' in Escherichia coli: The cause of extra mass of 258 or 178 Da in fusion proteins. *Anal. Biochem.* **267**, 169–184 (1999).
