# Supplementary material for: Competitive protein recruitment in artificial cells
Source: Commun Chem. 2024 Jun 28;7:148. doi: 10.1038/s42004-024-01229-9 (PMC11213860; doi:10.1038/s42004-024-01229-9)
Supplement: Supplementary file 3 — Description of Additional Supplementary Files [file 42004_2024_1229_MOESM3_ESM.pdf]

Description of Additional Supplementary Files

File name: Supplementary Data 1

Description: NMR spectra

File name: Supplementary Data 2

Description: Source data for main text Figures
