# Supplementary material for: Competitive protein recruitment in artificial cells
Source: Commun Chem. 2024 Jun 28;7:148. doi: 10.1038/s42004-024-01229-9 (PMC11213860; doi:10.1038/s42004-024-01229-9)
Supplement: Supplementary file 4 — Supplementary Data 1 [file 42004_2024_1229_MOESM4_ESM.docx]

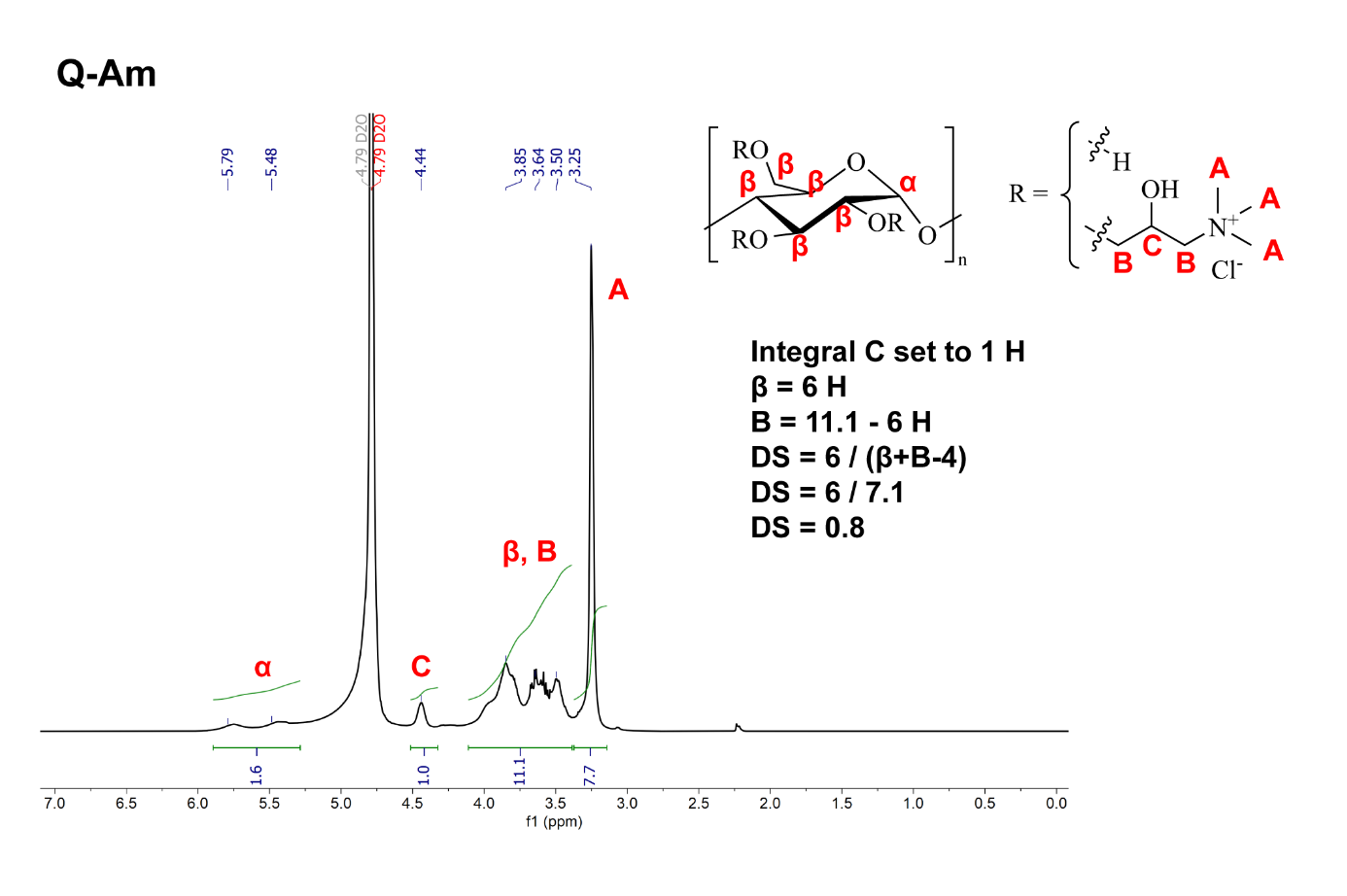


^1^H NMR spectrum (400 MHz) of Q-Am in D_2_O, with calculation of the degree of substitution.


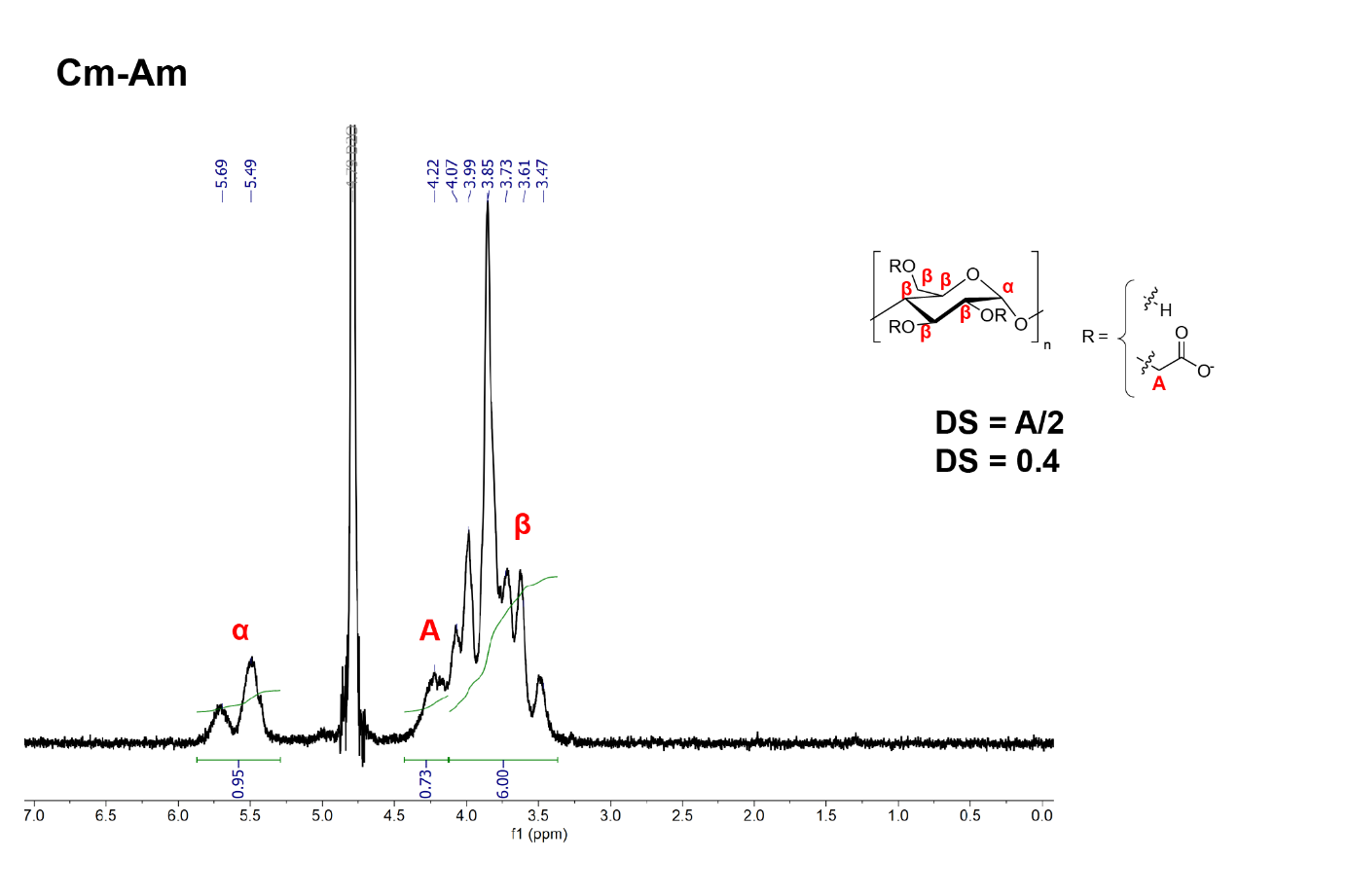


^1^H NMR spectrum (400 MHz) of Cm-Am in D_2_O taken, with calculation of the degree of substitution.


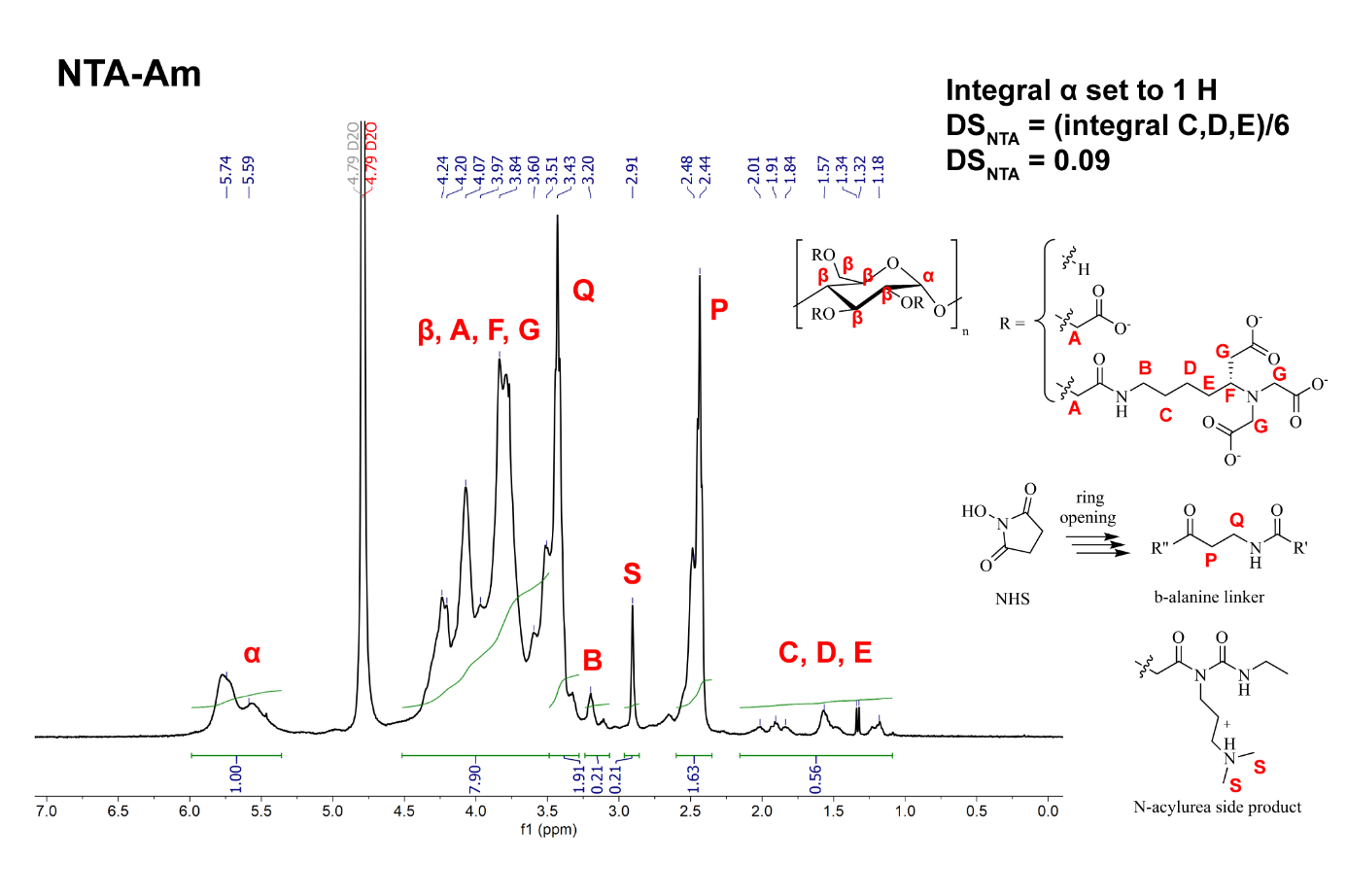


^1^H NMR spectrum (400 MHz) of NTA-Am in D_2_O, with calculation of the degree of substitution.


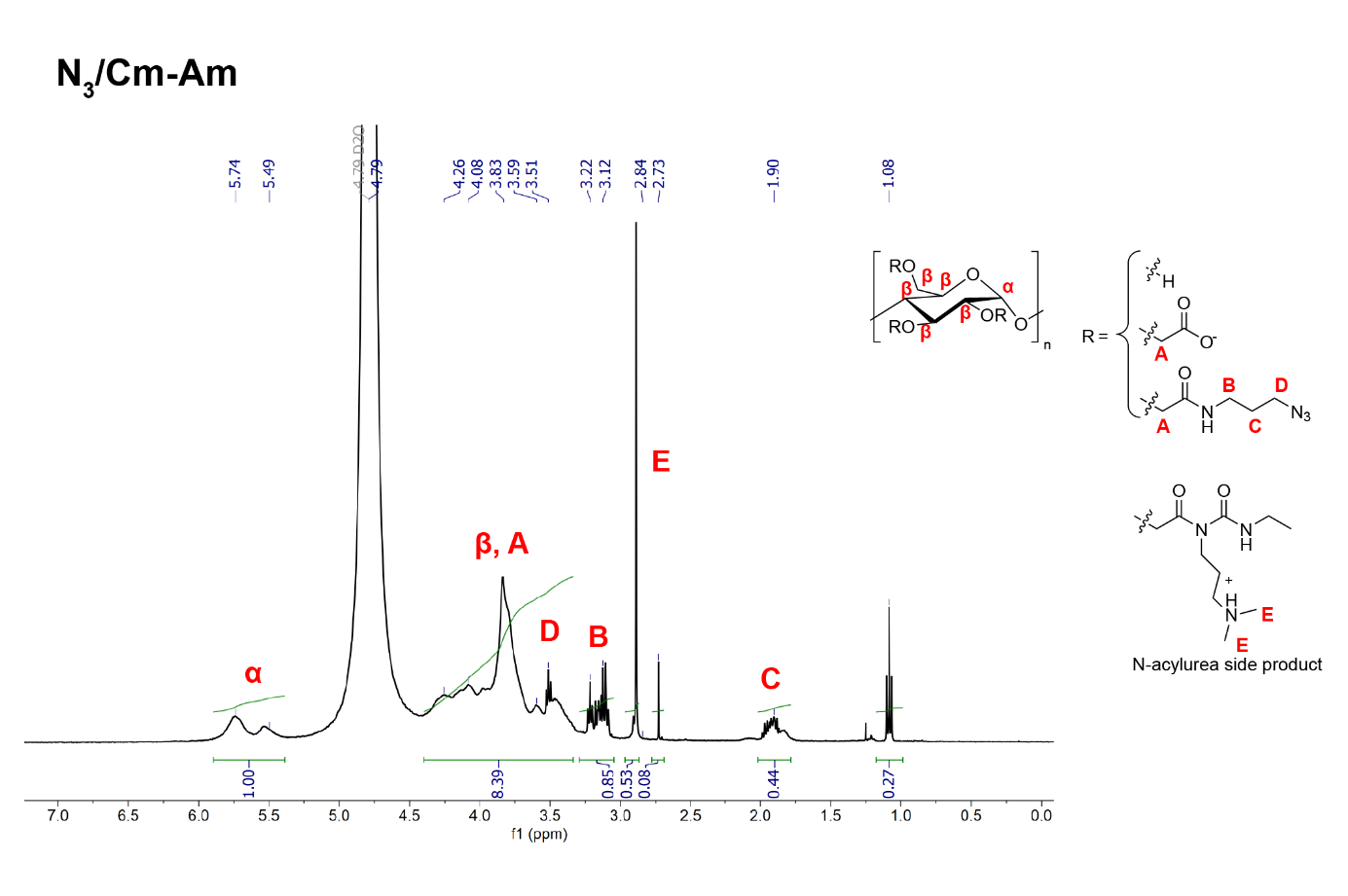


^1^H NMR spectrum (400 MHz) of N_3_/Cm-Am in D_2_O.


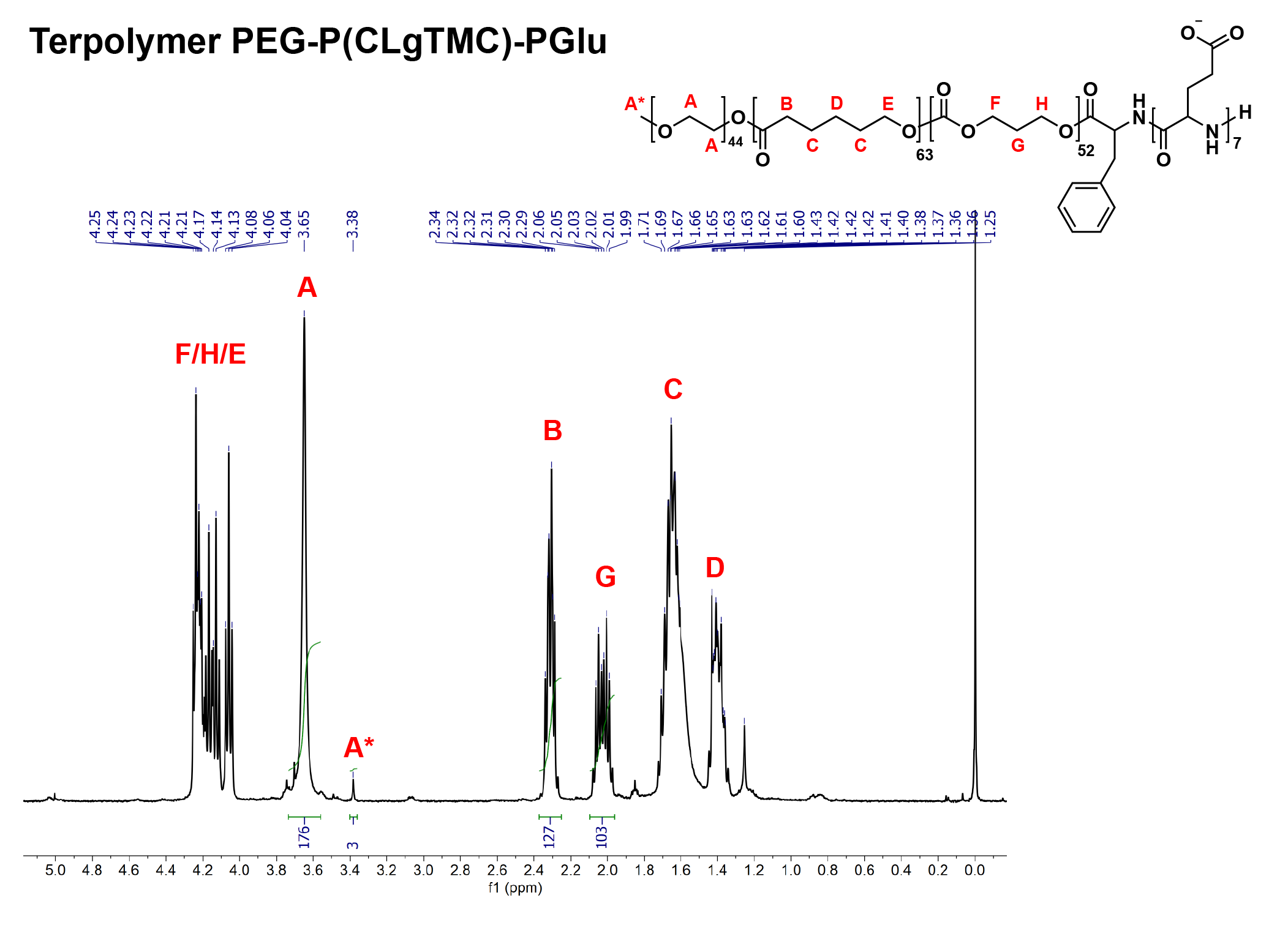


^1^H NMR spectrum (400 MHz) of terpolymer in CDCl_3_.
